# Spatial structure triggers systematic foraging: Segmenting search displays leads to searching by segments

**DOI:** 10.3758/s13414-026-03287-8

**Published:** 2026-06-09

**Authors:** Peter J. Goodwin, Filipe Cristino, Duncan Guest, Christina J. Howard

**Affiliations:** Department of Psychology, Nottingham Trent University, 50 Shakespeare Street, Nottingham, UK

**Keywords:** Visual search, Visual attention, Spatial cognition

## Abstract

Spatial structure often provides information about the likely location of targets in visual search tasks and in real life. However, the extent to which spatial structure affects attention when it provides no information about target locations is less well understood. This is investigated here using a visual foraging task, whereby individuals search for many targets amongst many distractors. In six online experiments, participants foraged for multiple instances of two target types amongst two distractor types, defined by either features or conjunctions. Experiments comprised a series of manipulations of the presence, quantity, visibility, and availability of spatial structure. This was achieved via visibly segmenting displays into areas containing equal numbers of objects. These experiments assessed the effects of spatial structure on foraging using a broad range of measures and across variations including the presence of time limits and visual feedback within the display. Task-irrelevant spatial structure consistently encouraged systematic foraging, whereby participants were more likely to continue foraging within one display segment rather than to move to another. Foraging in this manner was associated with costs to foraging efficiency, and benefits to spatial memory. The general strength of the visual information about spatial structure modified the extent to which this information affected systematic foraging and foraging efficiency. Overall, spatial structure can affect individuals’ foraging patterns and strategies even when it provides no information about the potential locations of targets.

**Supplementary Information:**

The online version contains supplementary material available at 10.3758/s13414-026-03287-8.

## Introduction

Remember the last time you were running late and were looking for your phone, keys, and money? As humans, we often need to search for more than one thing at a time. To do this effectively, we need to process information about the scene that we are searching such as its spatial layout and the likely locations of target objects. The overall spatial envelope of scenes is rapidly processed by the visual system (Oliva & Torralba, 2001) and the overall ‘gist’ is quickly assessed (Oliva, 2005), containing information about the overall physical and semantic properties of a scene. For example, one glance through an unfamiliar window might be enough for us to determine that this is an open green space or a busy street scene. Various paradigms have offered insights into such processes of scene perception and visual search. The focus of the work presented here is to isolate and assess the role of spatial structure on the dynamics of attention within the context of visual foraging.

One of the most well-developed models of visual search is that of Guided Search (Wolfe, 1994, 2021). Within Guided Search 6.0 (Wolfe, 2021), spatial structure is embedded within the priority map for attention and is inherently spatial in nature. For example, guidance from bottom-up low-level features is realised through prioritised locations in the priority map. Scene semantics and syntax are likewise embedded in spatial contexts, for example, searching for a fork on a kitchen counter in preference to looking for it above a window is a process underpinned by, amongst other things, spatial processing. In classic visual search tasks, attention can be cued to spatial locations such as surfaces (e.g., Wolfe et al., 2009) or depth planes (Roberts et al., 2015) when these are informative as to the location of the target. What is less clear is how spatial structure influences search when it contains no information as to the location of targets.

A handful of studies have investigated the role of spatial structure in search where that structure is not informative of target locations. For example, Treisman (1982) reported that clustering objects in a search display affected conjunction but not feature searches. For conjunction search, response times were consistent with attention visiting each cluster serially.

Nakashima and Yokosawa (2013) used segmenting lines to manipulate the apparent visual-spatial structure of search arrays. Participants performed a single-target search in either efficient (target C amongst distractor Os) or inefficient (target O amongst distractor Cs) conditions, with varying numbers of divided subsections within the display. This segmentation of the display was observed to slow performance during the efficient search task but facilitated inefficient search. Nakashima and Yokosawa (2013) suggest these segmenting lines may interfere in early visual search processes, thereby slowing down otherwise rapid search. Later in the search these lines may have provided grouping cues to facilitate search strategy in some way such as spatially structured search, though this was not directly investigated.

There is some evidence that individuals tend to adopt spatially systematic strategies to complete searches for a single target. Gilchrist and Harvey (2006) showed that when participants searched grid-like displays of objects, they tended to exhibit more horizontal than vertical eye movements, indicative of row-by-row scanning. This pattern existed even when items were removed from the display to make it less grid-like. Therefore, the spatial structure provided by this grid-like display may have promoted this apparent spatially systematic search strategy. In applied contexts, where there are clear implications for missing targets, there appear to be somewhat mixed findings regarding the utility of spatially systematic search strategies. Training medical students to use a spatially systematic search strategy improves detection of pulmonary nodules (abnormalities) in chest radiology (Auffermann et al., 2016). Radiology expertise is associated with increased use of systematic scanning of medical images, though the relationship between the use of this systematic scanning and performance is not straightforward (Kok et al., 2016). Therefore, in traditional and more applied visual search tasks, although it is somewhat unclear whether spatial systematicity aids in search, there are indications that spatial structure may in some circumstances trigger the adoption of spatially systematic search strategies.

Research investigating human foraging behaviours typically involves tasks that require a greater degree of spatial exploration, for example either by using a larger scale display, by necessitating more motor engagement with array locations or by having participants search for many instances of one or more target types in the display. It has been shown that people appear to adopt spatially systematic search strategies in large-scale open terrain-based search tasks (Riggs et al., 2017) and in searches requiring walking around a search display and pressing buttons on the floor (Pellicano et al., 2011). De Lillo et al. (2014) showed that participants demonstrate spatial systematicity when exploring locations in virtual reality (VR) space. Kerster et al. (2016) also report spatially systematic behaviours during foraging. In their task, participants made mouse clicks within a blank display and received feedback when they clicked on a target. They found that participants demonstrated spatial systematicity by tending to cluster their mouse-clicks around areas that had already yielded targets. Tagu and Kristjansson (2022) examined the effect of clustering targets of higher reward value in ‘patches’ in a task requiring foraging for many instances of multiple target types. However, the presence of spatial structure in this study was always combined with a clustering of targets with higher reward value, making it difficult to draw direct conclusions about the effects of spatial structure manipulated independently of other factors.

Kristjansson et al. (2014) introduced a visual foraging task in which participants search an array (e.g., by tapping on a screen) for many instances of targets. Targets can be defined by one feature or a conjunction of features. This foraging paradigm can produce a rich dataset, as demonstrated by the range of ways it has been used to show differences between feature and conjunction foraging activity (see Kristjánsson et al., 2019). In conjunction foraging trials, foraging routes are less optimal, trial durations are longer, and foraging accuracy is lower than is the case for feature foraging trials (Jóhannesson, et al., 2017; Kristjánsson et al., 2014; Thornton et al., 2019). One key difference between feature and conjunction foraging is how frequently people change the type of target they are looking for. Individuals frequently switch between the types of targets they select when in feature foraging and make far fewer switches in conjunction foraging (Jóhannesson et al., 2016, 2017; Kristjánsson et al., 2014, 2018, Kristjánsson, Björnsson et al., 2020a, Kristjánsson, Thornton et al., 2020b; Ólafsdóttir et al., 2019; Thornton et al., 2019). Furthermore, this paradigm requires continuous, temporally extended exploration of the foraging display and does not end after a target is found, making it ideal for investigating the ongoing dynamics of attention.

Spatial structure appears to impact efficient and inefficient visual search differently (Nakashima & Yokosawa, 2013). Similarly, efficient (feature) and inefficient (conjunction) foraging both exhibit key differences in duration, route optimality, accuracy, and target switches (Kristjánsson et al., 2019). Therefore, understanding how spatial structure interacts with such differences within a foraging context is important for a number of reasons. First, foraging will normally occur within a spatial context of some kind, and so understanding how a spatial context impacts foraging is important. It might be that bottom-up spatial aspects of the display over-ride typical preferences for how we forage in feature foraging or conjunction foraging displays. In so doing it might impact foraging efficiency. Second, any interaction between the imposition of spatial structure and foraging behaviour in efficient/inefficient foraging may help shed light on some of the processes that underpin foraging.

The present work therefore sought to investigate the extent to which foraging is conducted in a spatially systematic manner, and the effect of imposing non-informative spatial structure on this foraging process. Across six experiments, the present work explores the influence of spatial structure on such search for multiple targets. Spatial structure was manipulated via imposing and removing segmenting lines onto feature and conjunction foraging displays of 40 targets and 40 distractors. First, we expected to replicate known differences between feature and conjunction foraging processes. Second, we expected that in the presence of visible spatial structure, participants would adopt a spatially systematic search strategy, evidenced by a reduction in the frequency of switching between segments of the display.

In Experiment 1, the effects of display segmentation on foraging for feature and conjunction targets were assessed. Experiment 2 examined how increasing the amount of segmentation in the display affects foraging behaviours and the decisions involved in doing so. Experiment 3 is an investigation into the effects of imposing time limits on foraging and effects of segmentation. Experiment 4 assessed the effects of segmentation with fainter segmenting lines, under time limited conditions. In Experiment 5, segmenting lines vanished after being previewed. Finally, Experiment 6 assessed effects of segmentation on foraging when displays do not contain information about search history (targets did not disappear after selection). Together these experiments provide an overview of how spatial structure, here in the form of display segmentation in various forms, affects the ways that individuals forage displays for multiple targets.

### Experiments 1–6: Methodology

The common methodology of all six studies is given below, with any departures from this methodology stated for each experiment.

#### Participants

In all experiments, participants were asked to wear their glasses, or contact lenses, if they normally used these whilst working at a computer. Individuals with atypical colour vision or neurological disorders were asked not to participate. The research was approved by the Nottingham Trent University College Research Ethics Committee.

#### Design

Unless otherwise stated, each experiment used a within-groups factorial design with factors of segmentation type (2 levels: Non-segmented display and Segmented display) and search type (2 levels: Feature foraging and Conjunction foraging). Experiments were presented in a blocked design, comprising four blocks: one block for each of the four experimental conditions. Blocks were presented in a randomised order and contained four trials each. Each experiment began with one feature foraging and one conjunction foraging practice trial using non-segmented displays.

#### Materials

All experiments occurred online and were presented on participants’ own devices. A Qualtrics questionnaire included questions on participants’ age, gender, and device used. All experimental code was written in Python, using Psychopy (Peirce et al., 2019), and JavaScript. The experimental task was hosted on Pavlovia.org and was accessed via a link in the Qualtrics questionnaire. Questionnaire templates and experiment scripts are provided alongside the experimental data at: https://osf.io/6acm9/?view_only=f922f7ad07624bc181a4d9987f371ded.

All experimental trials involved a foraging array of 80 coloured objects. Foraging displays contained 80 objects: 40 targets and 40 distractors. In feature foraging trials, targets comprised 20 red and 20 green circles, whereas distractors comprised 20 blue and 20 yellow circles. In conjunction foraging trials, targets comprised 20 red circles and 20 green squares, whereas distractors comprised 20 green circles and 20 red squares. This is in line with the manipulations presented by Kristjánsson et al. (2014). Experimental displays were normalised using Psychopy so that displays always remained square regardless of screen size and aspect ratio.

**Fig. 1:**
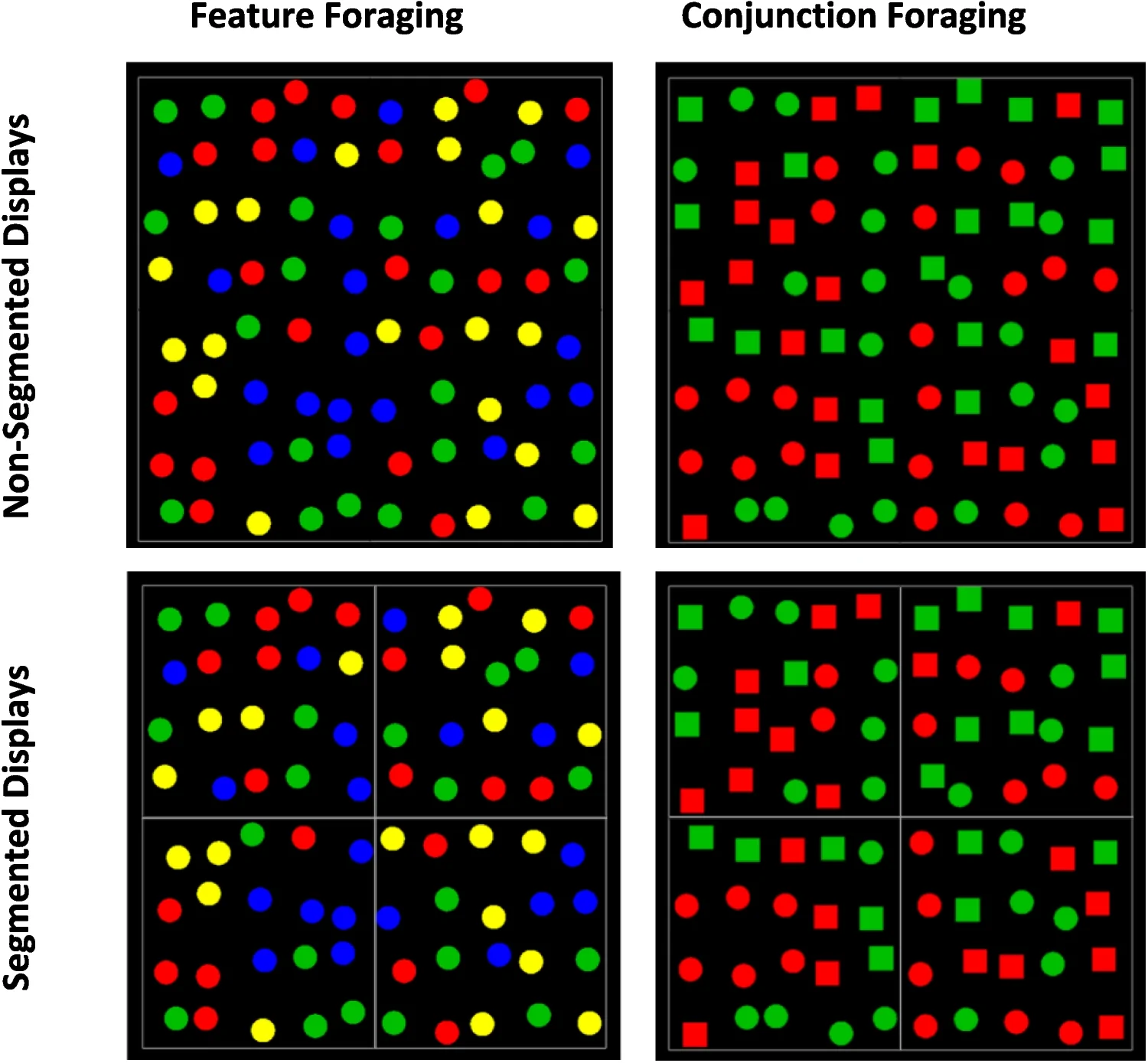
Examples of displays. In feature foraging trials, targets comprised red and green circles. In conjunction foraging trials, targets comprised red circles and green squares

Objects were presented against a black background and contained within a square border measuring 0.9 x 0.9 Psychopy normalised units. This border was offset from the top and bottom of the screen by 0.05 units**.** During segmented display trials, the array was divided into four equally sized square segments containing 20 objects each. Unless otherwise stated, bordering and segmenting lines were mid-grey (RGB: [125,125,125]). All objects had a height and width of 0.045 normalised units. Object positions were first randomly allocated across an 8x10 (invisible) grid. Objects were then randomly jittered around this grid position by a horizontal factor of ±0.02 units and a vertical factor of ±0.025 units and never overlapped each other or with any segmenting lines in the display. Examples of displays are shown in Fig. 1.

#### Procedure

Participants were provided with a URL link to the experiment to complete using their own device in their own time. Note that participants were instructed not to use a mobile device to complete the task. Those that did use a mobile device had their data removed from any analyses. Participants first completed a short Qualtrics questionnaire and were then directed to the experimental task via a URL link. After completing the experimental task, participants were redirected to the questionnaire and asked about the strategies they used in the task. Finally, participants were debriefed. Experiments in which participants were not recruited via Prolific.com either received credits that could grant use of the university’s participant recruitment systems, else were otherwise asked for a contact email address and entered £50 Amazon voucher prize draw.

At the start of the experimental task, participants were instructed that they would need to find and click on all the targets they saw on their screen as quickly and accurately as possible whilst avoiding distractors. Participants were informed that each trial would have two types of targets for them to find, and they should find all occurrences of these two target types to progress to the next trial. To facilitate understanding, participants were shown a diagram that explained the task and completed two practice trials. Participants then completed the 16 experimental trials. Each trial began with a display indicating the two types of foraging targets. Participants then clicked a centralised start button which triggered the foraging display to appear. They then foraged for targets by clicking them. Targets, but not distractors, disappeared from the display when clicked. Note that the trial continued if distractors were clicked and only ended when all targets had been selected (except for experiments with time limits whereby trials ended after 20 s if any targets had not been selected). Trial blocks were separated by a break screen informing participants of their progress (25%, 50%, or 75% complete). Participants could take a break on these screens and only click to continue when ready.

#### Analysis

Data from the six experiments presented here can be found in the Open Science Framework (OSF) repository at: https://osf.io/6acm9/?view_only=f922f7ad07624bc181a4d9987f371ded. Data were cleaned and analysed in R (R Core Team, 2025). Two-way repeated-measures ANOVAs to examine how factors of search type (feature and conjunction foraging task) and segmentation type (non-segmented and segmented displays) affected each measure. ANOVAs were conducted using the Afex package in R (Singmann et al., 2015) Post hoc tests were carried out using the emmeans package in R (Lenth, 2023, v1.8.7), to explore effects of segmentation and search type where appropriate. Where measures did not meet the assumption of equal variance across groups (as tested by Levene’s test for equality of variance, using the car package in R (Fox & Weisberg, 2019), additional non-parametric analyses were conducted (a Friedman test with post hoc tests conducted using a Wilcoxon signed-rank test with a Bonferroni correction applied, both within the R package rstatix, Kassambara, 2019). However, ANOVA results are present throughout for completeness regarding effects and interactions. Although some measures do not meet assumptions of normality and equivalence of variances, understanding how search and segmentation types interact in foraging is of theoretical importance. This is not possible using only non-parametric equivalents described above. Furthermore, the ANOVA method is argued to be robust in cases where such assumptions are violated (e.g., Blanca et al., 2023). For the reasons given here, Experiments 1–6 contain both parametric and non-parametric statistical analyses.

## Experiment 1

In Experiment 1, participants foraged either segmented or non-segmented displays for either 40 feature-defined or 40 conjunction-defined targets. Trials ended when all targets had been selected. Targets vanished after selection.

### Data cleaning

A total of 131 participants were recruited for Experiment 1 from the NTU undergraduate population and the general public. Of these, 29 were excluded for not meeting the recruitment criteria (i.e., using a touch screen device or entering an incorrect ID code, being aged > 59 years). This left a sample of 102 participants (86 F, 17 M) aged 18–56 years (mean = 23.56 years, *SD* = 9.75 years) from an undergraduate population and the general public. Power analyses estimated a required sample size of 27 participants; however, these estimates were based on laboratory-based research. The present research used a liberal sample as it was unknown how using online methods and involving display segmentation might influence findings. Additionally, a post hoc power analysis conducted in G*Power (Faul et al., 2007) on the smallest significant effect size in Experiment 1 (*η*_*p*_^*2*^ =.05) found that a sample of 102 participants gave a statistical power ≥.999.

Previous visual foraging work has excluded trials containing distractor selections from analyses (e.g., Kristjansson et al., 2014). Therefore, with the exception of analyses of foraging accuracy, trials containing errors (in which participants selected distractors) were removed from the analysis (14.44% of datapoints). Additionally, to remove anomalous data and extreme data affected by issues relating to participants’ devices, data outside of 3 standard deviations of the mean was excluded for each dependent variable. Note that for a statistical power of >.80, a sample of 28 participants was required. Therefore, even with this data exclusion, Experiment 1 had sufficient statistical power to detect meaningful effects.

### Results

#### Foraging accuracy

**Fig. 2:**
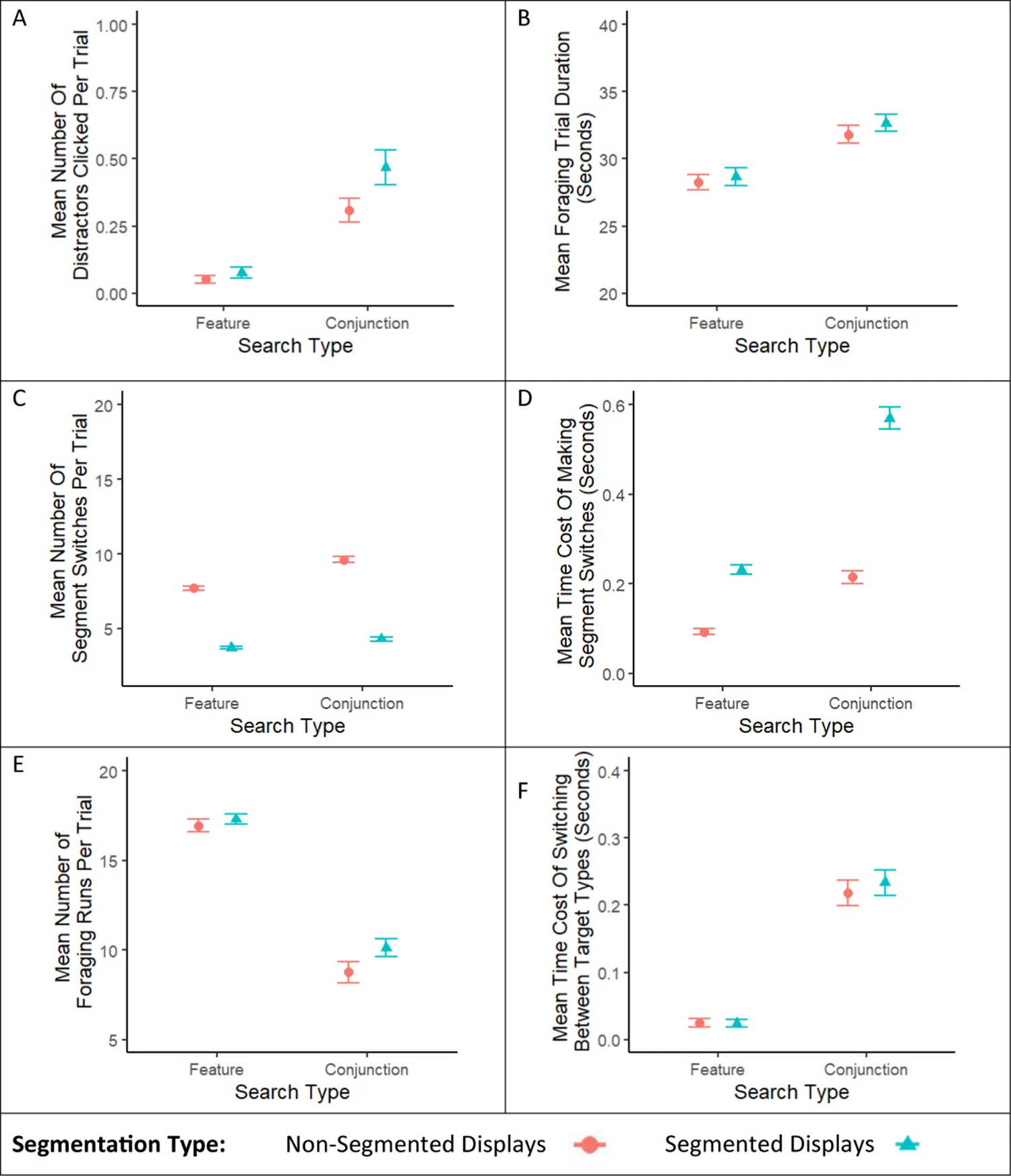
A–F Effects of search and segmentation type on (**A**) foraging accuracy, (**B**) trial durations, (**C**) segment switches, (**D**) segment switch costs, (**E**) foraging runs*, *and (**F**) target type switch costs. Points represent mean values, and error bars represent standard errors

Foraging accuracy was measured using the number of times participants erroneously clicked on a distractor: fewer distractor clicks corresponds to more accurate foraging. Therefore a mean of 0.5, for example, would result if a participant clicked on zero distractors for half the trials and one distractor for the other half of trials. Note that trials otherwise excluded for containing errors were not excluded from the data in this analysis. Main effects of search type: *F*(1,93) = 50.05,* p <*.001, *η*_*p*_^*2*^ =.35, and segmentation type:* F*(1,93) = 8.95,* p =*.004, *η*_*p*_^*2*^ =.09, were found, as well as an interaction*, F*(1,93) = 4.67,* p =*.033, *η*_*p*_^*2*^ =.05. Post hoc comparisons showed significant differences between all conditions (*p*s <.04) except for between feature foraging trials involving segmented and non-segmented displays (*p* =.78). The measure of foraging accuracy did not meet the assumption of equal variances and so a Friedman ANOVA was conducted: χ^2^_F_(3) = 68.7,* p <.*001. Significant differences were detected in post hoc comparisons of all pairings (all* p <.*001) except for between segmented and non-segmented display trials in feature trials (*p* >.999) and in conjunction foraging trials (*p* =.06). Together these analyses indicate that more distractors were clicked in conjunction trials than feature trials, and this was even more prevalent in displays that were segmented. These effects are shown in Fig. 2A.

#### Foraging duration

Foraging duration was calculated as the length (in seconds) it took a participant to select all 40 targets in the display from the onset of the array. Main effects were found of search type*, F*(1,93) = 203.04,* p <*.001, *η*_*p*_^*2*^ =.69, and segmentation type*, F*(1,93) =10.44,* p* =.002, *η*_*p*_^*2*^ =.11, with no interaction*, F*(1,93) = 2.67* p =*.10, *η*_*p*_^*2*^ =.03. Segmentation was therefore associated with increased foraging duration, and foraging durations were also longer for conjunction trials relative to feature trials. These effects are shown in Fig. 2B.

#### Segment switches

A segment switch was defined as an event whereby a participant selected two successive targets from different segments of the display. This measure was calculated both for segmented trials in which dividing lines were visible and for non-segmented trials in which dividing lines were absent. Although dividing lines were not presented during non-segmented trials, one can still calculate the number of times a switch was made between quadrants that were identical to the segments. Whilst switches between quadrants are not segment switches per se as there was no visible segmentation in these trials, for ease of comparison hereafter we use the term segment switch to refer to switches between segments/quadrants (or equivalent where there are more than four segments). The number of switches between quadrants and the time taken to move between quadrants acts as an important baseline to understand whether segmentation has any impact on how the display is foraged and if moving between segments produces any time cost (referred to as a segment switch cost). Note that in non-segmented displays, although segmenting lines were not visible, a segment switch was identified as having occurred when successive target clicks crossed the invisible segmenting lines that were made visible in the segmented displays. Whilst these lines were also the vertical or horizontal midline of the display, participants may or may not have been aware of these midlines. Importantly, all search behaviour can be characterised as a sequence of decisions about where to search next (e.g.*, *Findlay et al., 2001; Ludwig et al., 2005), therefore every movement around the display represents the outcome of such decision-making processes. As such assessing the extent to which foraging crossed between segments/quadrants and the time taken to do so provides an important indication as to the impact of segmentation. When displays were segmented, a minimum of three segment switches were needed to forage displays of four segments.

Main effects were found for factors of search type*, F*(1,93) = 102.60,* p <.*001, *η*_*p*_^*2*^ =.52, and segmentation type*, F*(1,93) = 928.96,* p <.*001, *η*_*p*_^*2*^ =.91. The two factors also interacted*, F*(1,93) = 33.07,* p <.*001, *η*_*p*_^*2*^ =.26, with post hoc comparisons of the conditions in this interaction all significantly differing (*p*s <.001). The measure of segment switch count did not meet the assumption of equal variances. Therefore, a Friedman ANOVA was conducted, χ^2^_F_(3) = 243,* p <.*001, with post hoc tests indicating significant differences detected between all pairs (*p*s <.001). As supported by the parametric and non-parametric analyses, the presence of segmentation was associated with a large impact on foraging behaviour, significantly reducing the number of segment switches. The number of segment switches was increased in conjunction trials relative to feature trials. This effect of search type was greater in non-segmented display trials than segmented display trials. These effects are shown in Fig. 2C.

#### Segment switch costs

The segment switch cost is the difference in the mean time taken to select two targets from the same segment versus two targets from different segments. Positive segment switch costs would reflect that participants took longer overall to select targets from different display segments than from within the same segment.

Main effects were found for factors of search type*, F*(1,97) =160.02,* p <.*001, *η*_*p*_^*2*^ =.62, and segmentation type*, F*(1,97) =211.46,* p <.*001, *η*_*p*_^*2*^ =.69. The two factors also interacted*, F*(1,75) = 40.37,* p <.*001, *η*_*p*_^*2*^ =.29. Post hoc comparisons of this interaction revealed significant differences between switch costs in all conditions (*p*s <.001). The measure of segment switch costs did not meet the assumption of equal variances, and so a Friedman ANOVA was conducted, χ^2^_F_(3) = 177,* p <.*001, with post hoc tests indicating significant differences detected between all pairs (*p*s <.001). Segmentation therefore led to increased switch costs, and switch costs were also greater for conjunction trials relative to feature trials. The effect of segmentation on switch costs was also greater in conjunction trials than feature trials. These effects are shown in Fig. 2D.

#### Foraging runs

Previous research using the foraging task used here has consistently found differences in the number of foraging runs made by participants (Kristjánsson et al., 2019). A foraging run involves selecting targets of the same type in succession, with runs ending when a target of a different type is selected (Kristjansson et al. 2014). There was an effect of search type*, F*(1,96) =241.77,* p <.*001, *η*_*p*_^*2*^ =.72, and segmentation type*, F*(1,96) =6.56,* p =.*01, *η*_*p*_^*2*^ =.06 but no interaction*, F*(1,96) = 1.27,* p =.*26, *η*_*p*_^*2*^ =.01. The measure of foraging runs did not meet the assumption of equal variances and so a Friedman ANOVA was conducted, χ^2^_F (_3) = 142,* p <.*001, with post hoc tests indicating differences detected between all pairs (*p*s <.001) except between segmented and non-segmented displays in feature (*p >.*999) and conjunction *(p =*.06) tasks. Segmentation therefore had no impact on the number of foraging runs, but more foraging runs were made during feature trials than conjunction foraging trials, as shown in Fig. 2E.

#### Target type switch costs

Target type switch costs reflect differences in the time taken to select two targets of the same type (e.g., two red circles) versus two different types (e.g., a red circle then a green circle). A positive cost reflects that it took longer to select targets of different types than of similar types.

For this measure, only a main effect of search type was observed, *F*(1,97) = 101.52,* p* <.001, *η*_*p*_^*2*^ =.51. No effect of segmentation type was observed, *F*(1,97) =.13,* p =*.72, *η*_*p*_^*2*^ =.001, nor an interaction of search and segmentation types*, F*(1,97) =.15,* p =*.70, η_p_^2^ =.002. The measure of target type switch costs did not meet the assumption of equal variances, and so a Friedman ANOVA was conducted, χ^2^_F_ (3) = 98,* p <.*001, with post hoc tests indicating significant differences detected between all pairings (*p*s <.001), except for between non-segmented and segmented displays in feature trials and conjunction trials (*p*s >.999). Segmentation therefore did not influence target type switch costs, but these costs were greater in conjunction trials than in feature trials, as shown in Fig. 2F.

### Discussion

Imposing a spatial structure had a clear impact on how participants foraged. This structure increased foraging duration, it also led to fewer segment switches, and it increased the time costs of making a segment switch. As such segmentation of the display appeared to make foraging behaviour less optimal. When foraging segmented displays, participants made near the minimum number of segment switches, suggesting that in the main they explored each segment near exhaustively before switching to another segment. When the display was segmented, moving between segments appeared to produce a time cost. This may be because top-down guidance was required to over-ride a salient boundary, or because the segmentation imposed an additional (and different) decision about when to switch between segments. Specifically, when segmenting lines were visible, participants may have to make a deliberate decision to switch between visibly delineated segments, potentially delaying the time to do so and contributing to longer foraging durations.

Consistent with previous findings, conjunction foraging trials took longer to complete and involved more errors and fewer foraging runs than feature foraging trials (Jóhannesson et al., 2016; Ólafsdóttir et al., 2020; Thornton et al., 2019). However, display segmentation did not affect feature and conjunction foraging equally. The impact of segmentation on error rates, segment switches, and segment switch costs was more pronounced for conjunction foraging trials than feature foraging trials. As conjunction search is characterized by inefficiency, it is possible that uncertainty about whether all the targets in a segment had been found made participants more cautious about choosing to switch segments, leading to more errors and increasing the segment switch cost.

Overall, the findings from Experiment 1 are surprising. Imposing spatial structure on the display changed foraging behaviour and made foraging less optimal. This runs counter to Nakashima and Yokosawa’s (2013) visual search findings. In their Experiment 1, the effect of display size was reduced for segmented displays and inefficient search, but this effect of segmentation was not seen for efficient search. In Experiments 2–3 they held display size constant and manipulated the number of segments (1, 4, 16, 64). For inefficient search segmenting the display into 4 increased search efficiency whereas for efficient search segmenting the display into 16 reduced search efficiency. In contrast, in our Experiment 1, imposing segmentation not only appeared to make foraging less efficient in both efficient (feature) and inefficient (conjunction) foraging, but inefficient foraging was particularly impacted. These contrasting findings highlight the importance of task differences. Foraging differs from search in that there are multiple target types and multiple instances of each target rather than just a single target and target type. In search, segmenting the display may therefore help add structure to an unstructured search pattern, aiding performance when search is inefficient. In foraging, as there is likely to be more than one target within a segment, segmentation appears to invoke a segment-by-segment foraging strategy where the choice to switch between segments produces a cost.

Given that segmentation appears to change foraging behaviour, Experiment 2 further explored the effects of segmentation by increasing the number of segments to eight. If segmenting creates additional decisions, such as when to switch between segments and which segment to switch to, then increasing the number of segments should increase this decisional load. It may also be the case that splitting a display up into four segments is something that participants see as useful and so use the segments to guide foraging. In comparison, splitting the display up into eight segments may not be seen as so useful, and so participants may display less apparently segment-by-segment foraging. Conversely, it might simply be that the spatial structure is so salient that it is difficult for participants to over-ride it, and so their foraging patterns may be dictated by the spatial structure of the display.

## Experiment 2

### Methods

Experiment 2 employed identical methods to Experiment 1, except that segmented displays were divided into eight sections of ten items each.

#### Sample

A total of 90 participants were recruited for Experiment 2, though 13 were excluded for not meeting the recruitment criteria (i.e., using a touch screen device or entering an incorrect ID code, being aged > 59 years). This left a sample of 77 participants (64 F, 12 M, one other, mean age = 20.5 years, *SD* = 3.22 years, age range: 18–29 years) from the undergraduate population and general public, although 90 participants were recruited in total. Post hoc power analysis in G*Power (Faul et al., 2007) found a sample of 77 participants gave a statistical power of ≥.999 for all significant effects in Experiment 2. Trials involving distractor selections were removed from the analysis (16.64% of datapoints). Data outside 3 standard deviations of the mean of each measure were also removed from the analysis to exclude anomalous data. A sample of 18 participants was required for a statistical power of >.80 in Experiment 2. The experiment therefore has sufficient statistical power to detect meaningful effects.

### Results

#### Foraging accuracy

**Fig. 3:**
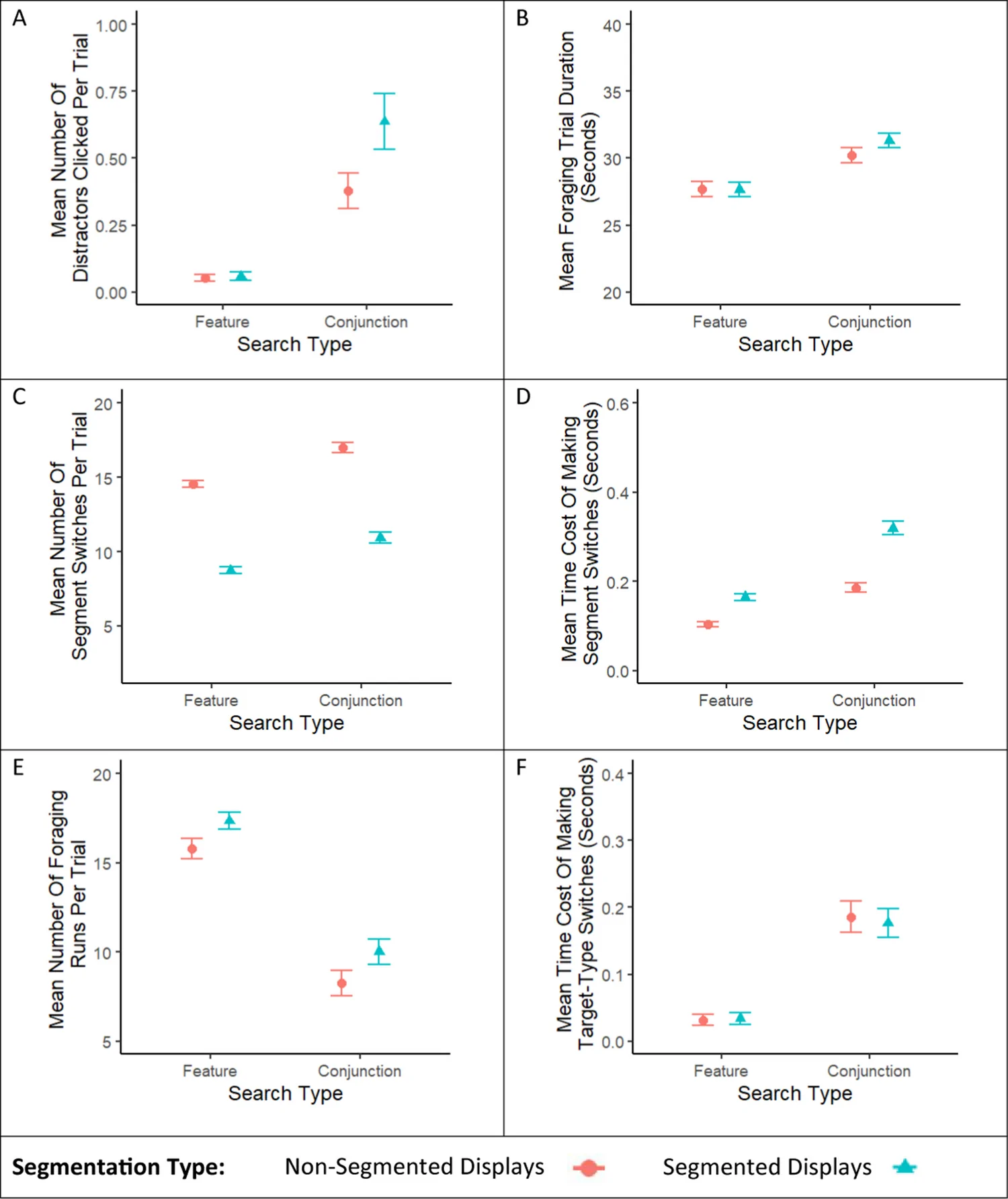
**A–F **Effects of search and segmentation type on (**A**) foraging accuracy, (**B**) trial durations, (**C**) segment switches, (**D**) segment switch costs, (**E**) foraging runs*, *and (**F**) target type switch costs. Points represent mean values, and error bars represent standard errors

Trials excluded for containing errors were included in the analysis of foraging accuracy only. Main effects of search type: *F*(1,71) = 42.20,* p <*.001, *η*_*p*_^*2*^ =.37, and segmentation type:* F*(1,71) = 9.59,* p =*.003, *η*_*p*_^*2*^ =.119, were found, as well as an interaction between these factors*, F*(1,71) = 9.16,* p =*.003, *η*_*p*_^*2*^ =.11. Post hoc comparisons showed differences between all conditions (*p*s ≤.01) except for the difference between segmented and non-segmented display trials in feature foraging *(p =*.998). The measure of foraging accuracy did not meet the assumption of equal variances and so a Friedman ANOVA was conducted: χ^2^_F_ (3) = 68.1,* p <*.001. Significant differences were detected in post hoc comparisons of all pairings (*p*s <.02) except for between segmented and non-segmented feature foraging trials (*p =.*999). As shown in Fig. 3A, the findings were similar to those observed in Experiment 1. Segmenting the display increased errors in conjunction foraging but not feature foraging. Additionally, conjunction foraging showed more errors for both segmented and non-segmented displays.

#### Foraging duration

For foraging duration, main effects were found of search type: *F*(1,67) = 141.26,* p <*.001, *η*_*p*_^*2*^ =.68 and segmentation type:* F*(1,67) = 6.62,* p =*.01, *η*_*p*_^*2*^ =.09, and an interaction*, F*(1,67) = 5.73,* p =*.02, *η*_*p*_^*2*^ =.08. As was the case in Experiment 1, foraging durations were greater in segmented displays and during conjunction foraging. Post hoc comparisons showed differences between all conditions (*p*s <.02) except for that between segmented and non-segmented feature foraging trials. Segmentation therefore appeared to increase foraging duration in conjunction foraging only (see Fig. 3B).

#### Segment switches

For segment switches (see Fig. 3C), there were main effects of search type*, F*(1,68) = 61.06,* p <.*001, *η*_*p*_^*2*^ =.47, and segmentation type*, F*(1,68) = 528.21,* p <.*001, *η*_*p*_^*2*^ =.89, but no interaction*, F*(1,68) = 0.24,* p =.*62, *η*_*p*_^*2*^ =.004. The measure of segment switch count did not meet the assumption of equal variances and so a Friedman ANOVA was conducted, χ^2^_F_ (3) = 156,* p <*.001. All post hoc comparisons were significant (*p*s <.001). As in Experiment 1, segmentation had an impact on foraging, resulting in fewer segment switches. Indeed, in a feature foraging task with a segmented display the mean number of segment switches was relatively close to the minimum number (seven) suggesting foraging behaviour in which segments tended to be foraged near exhaustively. More segment switches were also made in conjunction foraging trials.

#### Segment switch costs

For segment switch costs, there was a main effect of search type*, F*(1,66) = 99.62,* p <.*001, *η*_*p*_^*2*^ =.60, and segmentation type*, F*(1,66) = 71.96,* p <.*001, *η*_*p*_^*2*^ =.52, and an interaction*, F*(1,66) = 14.06,* p <*.001, *η*_*p*_^*2*^ =.18. Post hoc pairwise comparisons found differences between all pairings (*p*s <.001). The measure of segment switch cost did not meet the assumption of equal variances and so a Friedman ANOVA was conducted, χ^2^_F_ (3) = 102,* p <*.001. All post hoc comparisons were significant (*p*s <.001). As shown in Fig. 3D, segmentation increased the time taken to make a segment switch. Segment switches also took longer for conjunction foraging, and the impact of segmentation was particularly pronounced for conjunction foraging. The pattern of segment switch costs therefore seemed similar to that of Experiment 1. Interestingly, however, the size of the swich costs appeared to be reduced.

#### Foraging runs

For foraging runs (Fig. 3E), main effects were found for search type*, F*(1,68) = 131.99,* p <.*001, *η*_*p*_^*2*^ =.66, and segmentation type*, F*(1,68) = 13.50,* p <.*001, *η*_*p*_^*2*^ =.17, with no interaction,* F*(1,68) = 0.03,* p =.*86, η_p_^2^ <.001. Foraging runs did not meet the assumption of equal variances and so a Friedman ANOVA was conducted, χ^2^_F (_3) = 90.4,* p <.*001, with post hoc tests indicating differences between all pairs (*ps* ≤.008). Unlike Experiment 1, this effect of segmentation increased the number of foraging runs held both for parametric and non-parametric tests. It seems that increasing the number of segments led to a more robust impact on foraging runs. Like Experiment 1, more foraging runs were made during feature foraging relative to conjunction foraging.

#### Target type switch costs

For target type switch costs (see Fig. 3F), there was a main effect of search type*, F*(1,68) = 51.02,* p* <.001, *η*_*p*_^*2*^ =.43, but not segmentation type*, F*(1,68) =.08,* p =*.78, *η*_*p*_^*2*^ =.001, nor any interaction*, F*(1,68) =.31,* p =*.58, *η*_*p*_^*2*^ =.005. Target-type switch costs did not meet the assumption of equal variances, and so a Friedman ANOVA was conducted, χ^2^_F_(3) = 62.8,* p <.*001, with post hoc tests indicating differences detected between all pairings (*p*s <.001), except for between non-segmented and segmented displays in feature trials and in conjunction trials (*p*s >.999). As in Experiment 1, segmentation therefore had no impact on target type switch costs. Target-type switch costs were greatest in conjunction foraging, compared to feature foraging.

### Discussion

The findings from Experiment 2 further showed that the presence of spatial structure impacts foraging behaviour. As in Experiment 1, segmentation reduced the numbers of segment switches and increased errors, foraging duration and the segment switch cost. However, unlike Experiment 1, segmentation also increased the number of foraging runs. As was the case in Experiment 1, the effect of segmentation on errors and segment-switch cost were exacerbated in conjunction foraging. However, there were also some differences in the impact of segmentation on conjunction foraging between Experiments. In Experiment 2 a greater impact of segmentation on foraging duration in conjunction foraging was observed, whereas this was not the case in Experiment 1. In comparison, the greater impact of segmentation on segment switches in conjunction foraging that was observed in Experiment 1 was not observed in Experiment 2.

Overall, the results of Experiment 2 are similar to those of Experiment 1, with comparable mean values across most measures. The exceptions appeared to be segment switches and segment switch costs. As in Experiment 1, the introduction of segments tended to produce foraging behaviour characterised by searching near exhaustively within a segment. This was slightly less pronounced in Experiment 2 where there were eight segments, with conjunction foraging having a mean of around ten segment switches. Interestingly, the segment switch costs appeared to be reduced in Experiment 2 compared to Experiment 1. Increasing the decisional complexity of where to switch by increasing the number of segments did not appear to slow segment switches, suggesting this may not drive segment switch costs. As increasing the number of segments whilst maintaining the overall number of targets reduces the mean number of targets per segment, this may have increased participants’ confidence that they had foraged all targets in a given area, reducing the time taken to decide to switch.

As in Experiment 1, some effects of segmentation were heightened in the conjunction search, in all cases reducing the optimality of conjunction foraging. It appears that imposing spatial structure encourages more exhaustive foraging within segments, creating additional decisions regarding when to switch segment and to where. The effects of this additional decision may be magnified under more difficult circumstances, thus imposing costs on conjunction foraging.

Together, the findings of Experiments 1 and 2 demonstrate that spatial structure, even in the absence of semantic and syntactic information, has an impact on how individuals forage displays for multiple targets. Surprisingly, imposing a spatial structure evokes segment-by-segment foraging behaviour, which is less optimal. It is not clear, however, whether spatial structure encourages this adoption of a segment-by-segment strategy or whether factors such as salience of the structure are hard to ignore, making this foraging strategy hard to over-ride. Individuals may have adopted this strategy to organize their foraging as a cautious approach to avoid missing targets. If this is the case, imposing time limits on foraging might change how spatial structure affects foraging. That is, if the task is no longer to forage exhaustively, but rather to collect as many targets as possible, then there is greater emphasis on speed than on reducing the frequency of missing targets. Kristjansson et al. (2018) found that imposing short time limits on foraging changed how frequently individuals would otherwise switch between the types of targets they were foraging. Likewise, Thornton et al. (2022) showed that manipulating the pace at which foraging occurs also affected the rate of switches between target types. It has been shown therefore that time limits can affect foraging, but any interaction with effects of spatial structure is unknown. Therefore, the aim of Experiment 3 was to assess the effects of a time-limit on foraging segmented and non-segmented displays. Experiment 3 was identical to Experiment 1, but with a 20-s time limit on foraging.

## Experiment 3

### Methods

In Experiment 3, participants foraged segmented and non-segmented displays for as many of 40 feature- or conjunction-defined targets as possible. Trials ended after 20 s or when all targets had already been selected. The mean trial duration in Experiment 1 was 30.7 s, and so 20 s was a reasonable time to substantially forage displays, but insufficient time to find every target. Feedback was presented regarding how many targets had been selected on each trial.

#### Sample and data cleaning

Experiment 3 was pre-registered (https://osf.io/yhn2b), and a power analysis based on previous works and the results of Experiment 1 using G*Power (Faul et al., 2007) predicted that the sample size required was a minimum of 17 for power >.80. However, this was a very conservative estimate that was based on findings from studies that differed from the present one in aspects of time limits, online methods, and recruitment methods. Furthermore, non-parametric analyses were expected, error trials were expected to be removed, and the effects of time limits on spatial structure were not known, meaning that it was difficult to predict the precise sample size needed and the sample would need to be higher to compensate for these numerous factors. To be included, participants must:

1. Not complete the experiment on a touch screen device,
2. Not provide an in-use identification code,
3. Be aged between 18 and 59 years (to limit age effects; Madden, 2007),
4. Complete the experiment, and
5. Have three or more trials remaining after the removal of trials in which fewer than five targets were selected or a distractor was clicked.

A total of 105 participants were recruited via Prolific.co and compensated £2.50 for the 20-min experiment and 48 participants met the eligibility criteria used within Experiments 1 and 2. Because Experiment 3 employed a great level of time pressure for participants, it was piloted on 20 participants to confirm that participants recruited via Prolific.co would complete this type of speeded task. Seven of these pilot participants also met the data-inclusion criteria, and therefore were included in the main analysis alongside the 48 datasets since it is not ethical to exclude eligible data from analysis and reporting. The resulting 55 eligible participants (28 F, 27 M) were aged between 19 and 59 years (mean = 37.65 years, *SD* = 12.52 years). Any participants whose data were excluded because they selected distractors in multiple trials across one condition were only included in the analysis of foraging errors (distractor clicks). Post hoc power analysis conducted in G*Power (Faul et al., 2007) on the smallest significant effect size in Experiment 3 (*η*_*p*_^*2*^ =.14) found that a sample of 55 participants gave a statistical power ≥.999.

### Results

#### Foraging accuracy

**Fig. 4:**
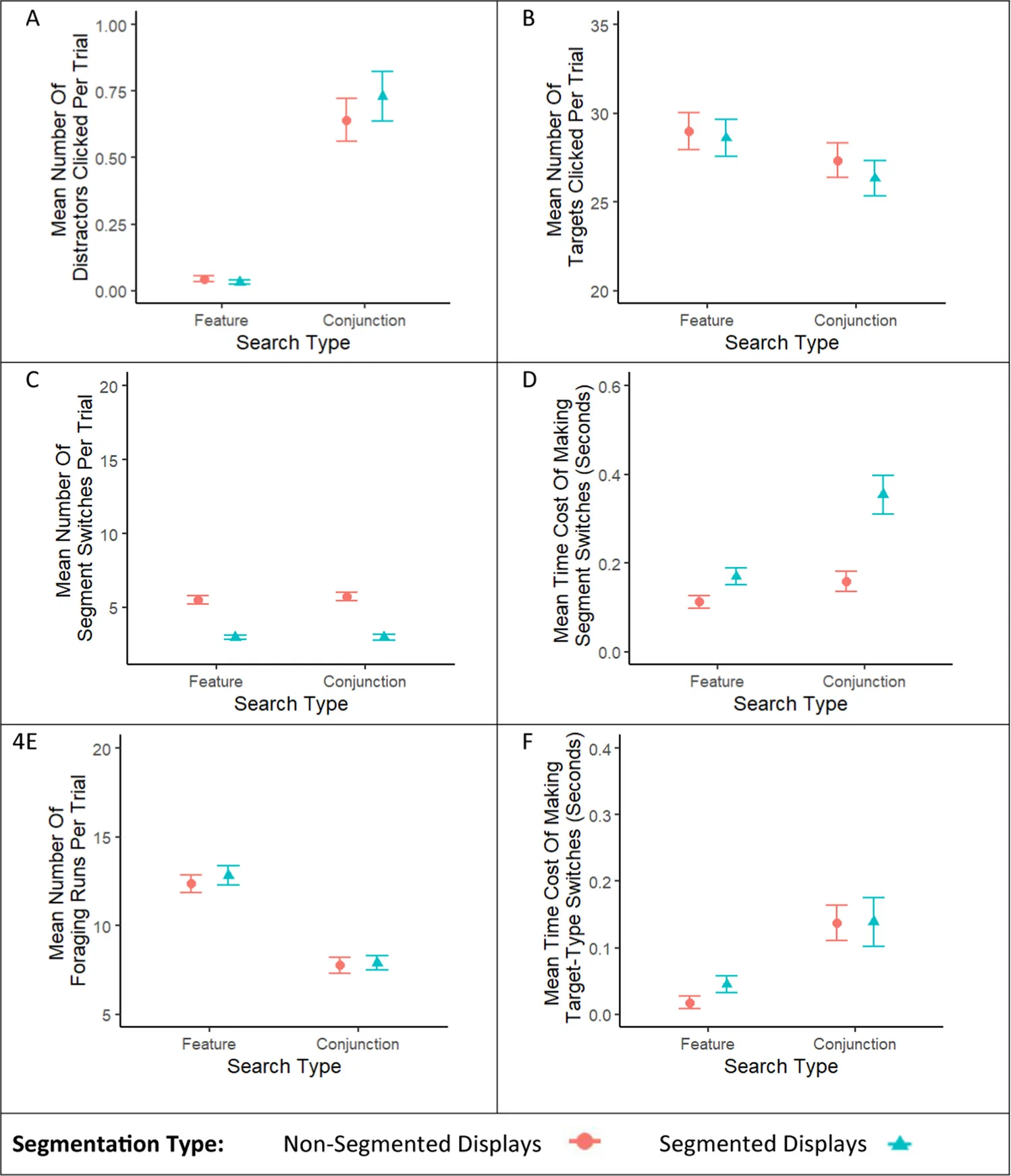
**A–F **Effects of search and segmentation type on (**A**) foraging accuracy, (**B**) number of targets selected, (**C**) segment switches, (**D**) segment switch costs, (**E**) foraging runs*,* and (**F**) target type switch costs. Points represent mean values, and error bars represent standard errors

A sample of 117 participants were included in the analyses of foraging accuracy (see Fig. 4A). This number includes the 55 participants who met the full data inclusion criteria, and 62 participants whose data were excluded only because they clicked on distractor objects in more than one trial within one experimental condition. An effect of search type was found*, F*(1,108) = 66.76,* p* <.001, *η*_*p*_^*2*^ =.39, but no effect of segmentation type*, F*(1,108) = 1.07,* p =*.30, *η*_*p*_^*2*^ =.001, nor an interaction*, F*(1,108) = 1.61,* p =*.21, *η*_*p*_^*2*^ =.01, was found. This did not meet the assumption of equal variances and so a Friedman ANOVA was conducted: χ^2^_F_ (3) = 141,* p <*.001. Differences were detected in post hoc comparisons of all pairings (*p*s <.001) except comparisons between segmented and non-segmented display trials for both feature and conjunction trials (*p*s =.999). More errors were made during conjunction than during feature foraging.

#### Number of target clicks

In Experiments 1 and 2, foraging time was defined as the time taken to select all targets. As Experiment 3 was time limited, we calculated the number of targets selected within the time limit (see Fig. 4B). There were main effects of search type*, F*(1,54) = 69.68,* p* <.001, *η*_*p*_^*2*^ =.56, and segmentation type*, F*(1,54) = 13.62,* p* <.001, *η*_*p*_^*2*^ =.20, and no interaction*, F*(1,54) = 2.46,* p =*.12, *η*_*p*_^*2*^ =.04. Fewer target clicks were made when segmentation was visibly present, versus when it was non-visible. Likewise, fewer target clicks were observed in conjunction foraging compared to feature foraging.

#### Segment switches

For segment switches, an effect of segmentation type was found*, F*(1,54) = 208.01,* p <.*001, *η*_*p*_^*2*^ =.79, but no effect of search type*, F*(1,54) = 1.04,* p =.*31, *η*_*p*_^*2*^ =.02, nor interaction (1,54) = 1.13,* p =.*29, *η*_*p*_^*2*^ =.02. Segment switch count did not meet the assumption of equal variances and so a Friedman ANOVA was conducted, χ^2^_F_ (3) = 126,* p <*.001. All post hoc comparisons were significant (*p*s <.001) except for the difference between feature and conjunction foraging in segmented displays and non-segmented displays (*p* ≥.999). Segmentation led to fewer segment switches as can be seen in Fig. 4C.

To examine whether the extent of segment switching was similar between Experiment 3 and the preceding two experiments, a normalized measure was created producing targets-per-segment-switch values. This took into account the fact that the number of target selections was lower in Experiment 3 due to the trial time limit and also that there were fewer targets per segment in Experiment 2 due to there being eight segments. This measure was calculated as the number of targets selected in total within a trial divided by a value given by the number of segment switches made plus 1. For example, in Experiment 1, if three segment switches were made to forage the four segments, since there were 40 targets, this normalised measure would have a value of 10 (i.e., average number of targets selected per segment switch). Additionally, because Experiment 2 featured eight segments, this normalised measure was doubled so that targets-per-segment-switch values were comparable to those in Experiments 1 and 3 where a display contained four segments.

A three-way ANOVA compared this normalised measure across Experiments (1, 2, and 3) by segmentation type (segmented vs. non-segmented) and search type (feature vs. display). There was no effect of experiment (*p* >.999), nor two-way interaction between experiment and either segmentation type or search type, nor three-way interaction (*p*s >.999). Therefore, there was no evidence that normalised targets-per-segment-switch values differed between Experiments 1–3, further suggesting that individuals foraged segmented displays in a similar manner across these three experiments.

#### Segment switch costs

For segment switch costs (see Fig. 4D), there were main effects of search type*, F*(1,54) = 20.24,* p <.*001, *η*_*p*_^*2*^ =.27, and segmentation type*, F*(1,54) = 25.98,* p <.*001, *η*_*p*_^*2*^ =.32, and an interaction*, F*(1,54) = 8.63,* p =*.005, *η*_*p*_^*2*^ =.14. Post hoc pairwise comparisons found differences only between the following pairings: conjunction non-segmented display trials versus conjunction segmented display trials; and feature segmented display trials versus conjunction segmented display trials (*p*s <.001). Segment switch costs did not meet the assumption of equal variances and so a Friedman ANOVA was conducted, χ^2^_F_ (3) = 44.5,* p <*.001. All post hoc tests were significant (*p*s <.03), except for the comparison of conjunction and feature trials involving non-segmented displays (*p* >.24). As in Experiments 1 and 2, segmentation appeared to increase segment switch costs, and this was driven by greater segment switch costs in segmented conjunction foraging.

#### Foraging runs

For foraging runs (see Fig. 4E), main effects were found for search type*, F*(1,54) = 79.95,* p <.*001, *η*_*p*_^*2*^ =.58, but not segmentation type*, F*(1,54) = 1.64,* p =*.21, *η*_*p*_^*2*^ =.03, nor their interaction*, F*(1,54) =.58,* p =*.45, *η*_*p*_^*2*^ =.01. The measure of foraging runs did not meet the assumption of equal variances and so a Friedman ANOVA was conducted, χ^2^_F_(3) = 85.2,* p <.*001, with post hoc tests indicating differences detected between all pairs (*p*s ≤.001), except for between non-segmented and segmented displays in feature trials *(p =*.43) and in conjunction trials (*p >*.999). Segmentation therefore had no impact on foraging runs in either parametric or non-parametric tests. As in Experiments 1 and 2, more foraging runs were made during feature than conjunction foraging.

#### Target type switch costs

For target type switch costs (see Fig. 4F), main effects were found for search type*, F*(1,54) = 28.71,* p <.*001, *η*_*p*_^*2*^ =.35, but not segmentation type*, F*(1,54) =.87,* p =*.36, *η*_*p*_^*2*^ =.02, and no interaction*, F*(1,54) =.13,* p =*.72, *η*_*p*_^*2*^ =.002. The measure of segment switch costs did not meet the assumption of equal variances, and so a Friedman ANOVA was conducted, χ^2^_F_(3) = 34.7,* p <.*001, with post hoc tests indicating differences detected between all pairings (*ps* <.002), except for between non-segmented and segmented displays in feature trials and in conjunction trials (*p*s >.999). Target type switch costs were greater during conjunction foraging than feature foraging, as was seen in Experiments 1 and 2.

### Discussion

The findings of Experiment 3 demonstrate that even under time-limited conditions, spatial structure can still impact foraging behaviour. This is shown most clearly in terms of the number of segment switches. When the display was segmented, participants made near-minimum segment switches for both feature and conjunction foraging, meaning they were unlikely to return to a segment once it had been left. As such, the time limit increased the extent to which foraging was completed in a segment-by-segment manner, even if there may have been potential benefits from performing the task differently. That this did not increase errors is likely because participants selected the targets they could find quickly within a segment and then switched segment, rather than foraging exhaustively within a segment. This idea is consistent with the fact that the mean number of foraging runs was lower in Experiment 3 than in Experiments 1 and 2. Switching segments still imposed a cost, and this cost was greater in conjunction foraging. As noted previously, this seems intuitive given that choosing to switch segment should be more difficult when the foraging task is more difficult, which is the case for conjunction foraging, which produced more errors and fewer target clicks.

Despite the task being time limited, the general patterns of findings in Experiment 3 look very similar to those in Experiments 1 and 2. This indicates a robustness of effects of segmentation and foraging task (feature/conjunction) to specifics of task parameters. In Experiment 3 where the number of targets clicked was calculated, the pattern of findings was remarkably similar to those of Experiments 1 and 2 where foraging duration was measured instead. This is perhaps counter-intuitive as it might seem the best strategy when foraging under time limits would be to focus on one target. Such a strategy would limit time-costs associated with changing the type of target being searched for. However, participants may have adopted a low-hanging fruit strategy and changed the type of target they foraged for whenever it took too long to find the targets they were previously searching for. Interestingly, this also potentially explains why the target-switch cost seemed reduced in conjunction foraging. Having a reduced threshold for switching targets would speed up the decision to switch, which would potentially have its greatest impact on tasks in which foraging is more difficult (i.e., conjunction foraging). If so, this would mean that the target-switch cost is not fixed, but malleable, and simply an artefact of a more difficult decision-making process.

As in Experiments 1 and 2, segmentation appeared to lead to less optimal foraging behaviour. In Experiment 3, the number of target clicks within the time limit was reduced for segmented displays. This suggests that even when the task is to forage as many targets as possible within a limited time frame, participants are either unable to ignore the spatial structure or still choose a strategy that uses spatial structure even if it leads to less optimal foraging. Additionally, the effects of time limits on search type match those of Experiments 1 and 2 where conjunction foraging took longer than feature foraging. Thus, when participants are allocated 20 s to carry out feature and conjunction foraging, as in Experiment 3, more targets were selected during feature foraging trials than conjunction foraging trials.

In Experiments 1–3 the spatial structure was clearly visible, and the salience of this structure may have triggered a perceptual grouping of items as segmented clusters, which is difficult to over-ride. Alternatively, the salience of the boundaries between regions may have made it effortful to cross them, with the effect that foraging was focused within a segment. To investigate this further, Experiments 4 and 5 assessed effects of structure with reduced visibility and availability, respectively.

## Experiment 4

### Method

In Experiment 4, the visibility of segmentation was reduced to investigate to assess subsequent effects of segmentation on foraging. The visibility of segmenting lines was reduced by changing their colour from mid grey [125,125,125] as in Experiments 1–3, to a lower contrast grey: [31,31,31]. All other elements of this experiment were identical to Experiment 3. It was expected that if segmenting lines were less salient this may reduce their impact on foraging behaviour.

#### Sample

Experiment 4 was pre-registered (https://osf.io/t75er) and in line with Experiment 3, which also time-limited, a final sample of 48 participants who met the inclusion criteria were recruited (also see Experiment 3). A total of 107 participants were recruited until data from 48 eligible participants had been collected. The 48 recruited Participants (33 F, 14 M, one unknown) were aged between 19 and 58 years (mean = 35.31 years, *SD* = 11.24 years). Of the 107 total participants recruited, those whose data were excluded because they selected distractors in multiple trials across one condition were included in the analysis of foraging errors (distractor clicks) only. All participants were recruited via Prolific.co and compensated £2.50 for the 20-min experiment. Post hoc power analysis conducted in G*Power (Faul et al., 2007) on the smallest significant effect size in Experiment 4 (*η*_*p*_^*2*^ =.10) found that a sample of 48 participants gave a statistical power ≥.999.

### Results

#### Foraging accuracy

Previously excluded participant data were also included in the analysis of foraging accuracy only (total *N* = 85: 49 F, 35 M, one unknown, mean age = 34.84 years, *SD* Age = 11.22 years, range = 19–58 years). These participants’ data were excluded from all other analyses for containing too many distractor clicks within trials, meaning that they did not meet the inclusion criteria for the remaining analyses.

**Fig. 5:**
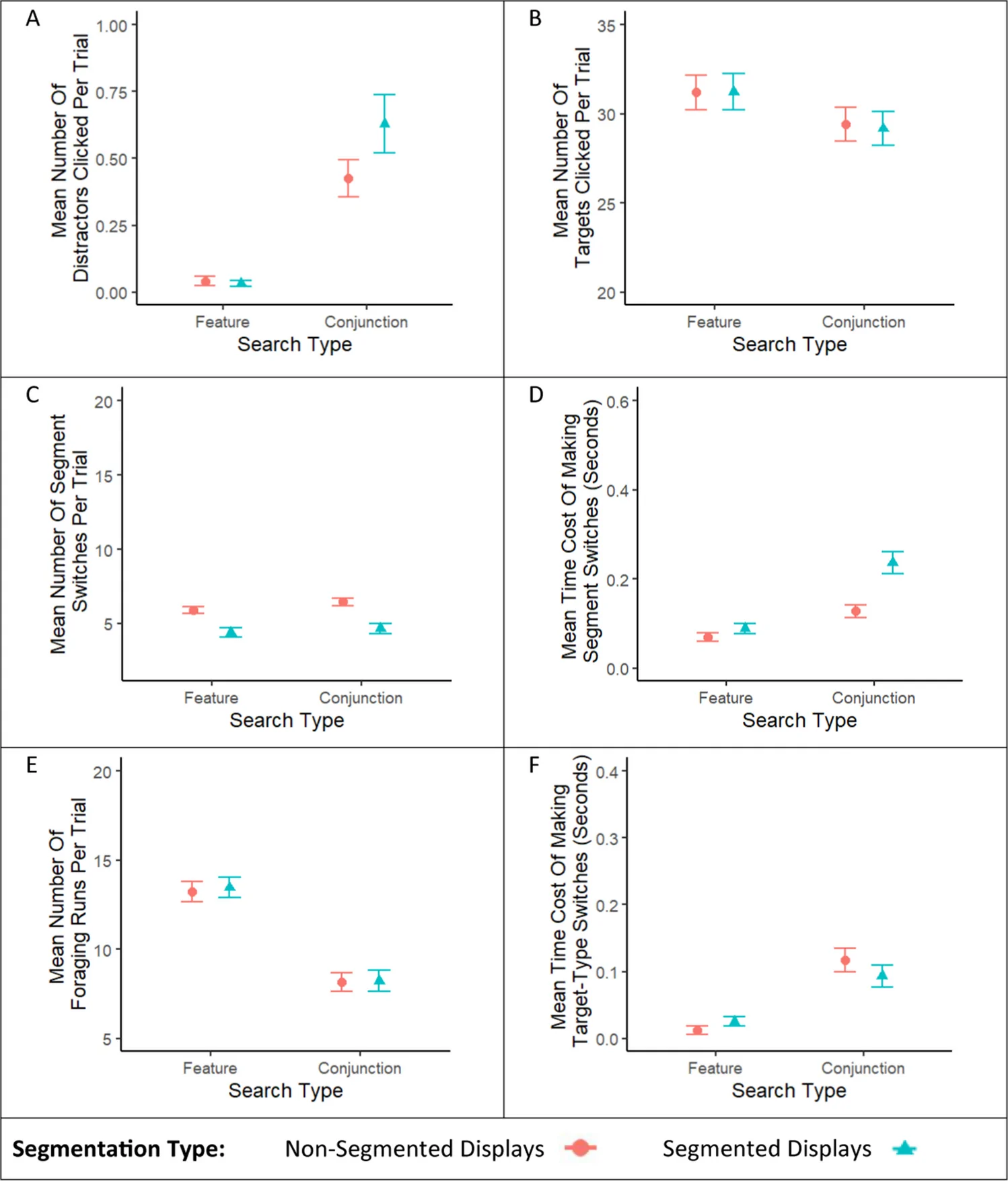
**A–F **Effects of search and segmentation type on (**A**) foraging accuracy, (**B**) number of targets selected, (**C**) segment switches, (**D**) segment switch costs, (**E**) foraging runs*,* and (**F**) target type switch costs. Points represent mean values, and error bars represent standard errors

There was an effect of search type*, F*(1,84) = 46.21,* p <*.001, *η*_*p*_^*2*^ =.35, but no effect of segmentation condition*, F*(1,84) = 3.89,* p =*.05 though there was an interaction*, F*(1,84) = 4.35,* p =*.04, *η*_*p*_^*2*^ =.05, with post hoc comparisons showing differences between all pairings (*p*s <.001) except for the comparison between segmented and non-segmented trials for feature foraging and for conjunction foraging (*p*s >.18). This measure did not meet the assumption of equal variances and so a Friedman ANOVA was conducted, which found differences between conditions: χ^2^_F_ (3) = 105,* p <*.001. All post hoc tests were significant (*p*s <.03), except for the comparison between segmented and non-segmented trials for feature foraging and for conjunction foraging (*p*s >.37). More errors were made during conjunction than feature foraging. These effects are shown in Fig. 5A.

#### Number of target clicks

For the number of targets selected (see Fig. 5B), there was an effect of search type*,F*(1,47) = 63.02,*p* <.001, *η*_*p*_^*2*^ =.57, but not of segmentation type*,F*(1,47) = 0.22,*p* =.64, *η*_*p*_^*2*^≤.001, nor any interaction*,F*(1,47) = 0.59,*p =*.44, η_p_^2^ =.01. More targets were selected during feature than conjunction foraging.

#### Segment switches

For segment switches (see Fig. 5C), there were effects of search type*, F*(1,47) = 8.57,* p =*.005, *η*_*p*_^*2*^ =.15, and segmentation type*, F*(1,47) = 35.58,* p <*.001, *η*_*p*_^*2*^ =.43, but no interaction*, F*(1,47) = 0.99,* p =.*33, *η*_*p*_^*2*^ =.02. Segmentation reduced the number of segment switches, with more segment switches made during conjunction foraging.

#### Segment switch costs

For segment switch costs (see Fig. 5D), main effects were found for search type*, F*(1,47) = 40.10,* p <.*001, *η*_*p*_^*2*^ =.46, and segmentation type*, F*(1,47) = 17.21,* p <.*001, *η*_*p*_^*2*^ =.27. These factors interacted*, F*(1,47) = 5.36,* p =*.03, *η*_*p*_^*2*^ =.10. Post hoc pairwise comparisons found differences between all pairings (*p*s <.03) except between segmented and non-segmented trials in feature foraging *(p =*.58). This measure did not meet the assumption of equal variances and so a Friedman ANOVA was conducted, χ^2^_F_ (3) = 38.1,* p <*.001. All post hoc comparisons were significant (*p*s <.05), except for between segmented and non-segmented display trials in feature foraging (*p =*.87). Overall, segment switch costs were greater during conjunction than feature foraging. In conjunction foraging trials, segment switch costs were greater in segmented than non-segmented displays, but not in feature foraging trials.

#### Foraging runs

For foraging runs (see Fig. 5E), only an effect of search type was found, *F(*1,47) = 80.86,* p <.*001, *η*_*p*_^*2*^ =.63. More foraging runs were made during feature foraging than conjunction foraging. There was no effect of segmentation type, *F(*1,47) =.26,* p =*.61, *η*_*p*_^*2*^ =.005, nor interaction, *F(*1,47) =.08,* p =*.77, *η*_*p*_^*2*^ =.002.

#### Target type switch costs

For target type switch costs (see Fig. 5F), there was a main effect of search type*, F*(1,47) = 56.33,* p <.*001, *η*_*p*_^*2*^ =.55 but not of segmentation type*, F*(1,47) =.17,* p =*.68, *η*_*p*_^*2*^ =.003, and no interaction*, F*(1,47) = 3.56,* p =*.07, *η*_*p*_^*2*^ =.07. The measure of target type switch costs did not meet the assumption of equal variances, and so a Friedman ANOVA was conducted, χ^2^_F_(3) = 72.6,* p <.*001, with post hoc tests indicating differences detected between all pairings (*p*s <.001), except for between non-segmented and segmented displays in feature trials and in conjunction trials (*p*s >.999). Target-type switch costs were greater overall during conjunction foraging than during feature foraging.

### Discussion

Across Experiments 1–3 segmentation led to less optimal foraging and effects on three or more of the foraging measures. In contrast, for Experiment 4, segmentation only impacted two foraging measures and did not impact the number of targets successfully clicked. Reducing the salience of the segmentation did appear to lessen the impact on foraging behaviour. Segmentation still encouraged foraging in a segment-by-segment manner with around four segment switches made in segmented trials. This was still greater than that seen in Experiment 3 however (mean ~three switches) suggesting that participants were slightly less bound to this approach. Interestingly, although segmentation still increased the segment switch cost in conjunction foraging, the size of the cost seemed slightly reduced relative to Experiment 3, suggesting that participants found it easier to switch segments.

As with any experimental design that randomizes experimental conditions, there will be a number of classic order effects that can influence how individuals complete tasks such as learning, fatigue, and carryover effects from exposure to different conditions Randomization is classically used to maximise the extent that these effects wash out, as we employed here. However, to check for the possibility that exposure to display segmentation could have affected foraging in subsequent non-segmented trials, we conducted further analyses on datasets from Experiments 1–4 in which participants happened to complete either both blocks of segmented trials first or both blocks of non-segmented trials first. The order of conditions did not affect trial duration (*p* =.30), the number of targets selected in a trial (*p* =.59), nor the number of segment switches made in a trial (*p* =.444). Although these experiments were not designed with the intention of exploring carryover effects, these tests reveal no evidence that exposure to one type of display (segmented or non-segmented) substantially carries over to the other later presented type of display.

It appears then that reducing the salience of the segments did reduce the impact of segmentation slightly. However, at the same time, it showed that even a faint segmentation line impacts foraging behaviour significantly, indicating this effect is very robust. It is also important to note the similarity of the patterns in the data across Experiments 1–4. This suggests that there is something about boundary lines between segments that appears to induce qualitatively different foraging behaviour. Indeed, the robustness of some of the effects suggests that this might be driven in a bottom-up manner, such that spatial structure is hard to ignore. We addressed this in Experiment 5, in which spatial structure was previewed on each trial but was not present during the trial.

## Experiment 5

Experiment 5 assessed the effects of segmentation on foraging behaviour when the segmentation was only pre-viewed before a trial and not visible during the trial. An absence of segmentation effects would indicate that the spatial structure needs to be present in order to impact foraging. Conversely, a segmentation effect would indicate that once a spatial structure is seen, its effects on foraging behaviour persist.

### Methods

Experiment 5 was identical to Experiment 1, with some key differences. Firstly, in each trial the foraging display was previewed for at least 1 s. After 1 s elapsed, a grey circle appeared in the center of the display. Participants could not select any targets from the display until they had clicked this circle. Importantly, in trials involving segmented displays, the segmenting lines within the display vanished immediately after clicking this circle. Segmenting lines were never visible during non-segmented trials. Therefore, segmented and non-segmented displays appeared identical during the foraging section of trials. In Experiment 5, segmentation was treated as a between-subjects factor rather than a repeated-measures factor, such that participants completed 16 trials involving exclusively either segmented, or non-segmented displays (eight feature- and eight conjunction-foraging trials) but never a mix of both segmentation types. Between-subjects designs, like repeated-measures designs, are classic experimental design types which can robustly assess effects of independent variables. For this experiment, segmentation was manipulated between-subjects to completely eliminate the possibility of carryover effects of exposure to segmentation on non-segmented trials. Effects of exposure to the preview on the active foraging that immediately follows the preview could be considered as a type of carryover effect during the relatively short time interval of a single trial duration and therefore it was important to avoid any contamination from carryover effects over the longer time interval between successive blocks. As such, data from Experiment 5 was analysed using mixed-factor ANOVAs whereby search type was treated as a within-groups factor and segmentation type was treated as a between-groups factor. Non-parametric tests were conducted using Kruskal-Wallis tests (R stats package – R Core Team, 2025), with Dunn’s test used for post hoc comparisons (R package rstatix – Kassambara, 2019), to account for the involvement of a between-group factor. To investigate whether differences in repeated-measures conditions (Feature vs conjunction foraging in segmented displays, and feature vs conjunction foraging in non-segmented displays) were significant, two additional Wilcoxon signed-rank tests with a Bonferroni correction applied were conducted. Unlike Experiments 3 and 4, Experiment 5 had no time limit.

#### Sample

A total of 118 participants were recruited for Experiment 5, though 21 were excluded for not meeting the recruitment criteria (i.e., using a touch screen device or entering an incorrect ID code). This left a sample of 97 participants (79 F, 17 M, one other, mean age = 21.35 years, *SD* = 5.30 years, age range: 18–55 years) from the undergraduate population and general public. Forty-one were in the segmented-previews group and 56 were in the non-segmented previews group. Although a total of 118 were recruited, post hoc power analysis in G*Power (Faul et al., 2007) found a sample of 97 participants gave a statistical power of ≥.999 for all significant effects in Experiment 5. As in Experiment 1, trials involving distractor selections were removed from the analysis (20% of datapoints). Data outside three standard deviations of the mean of each measure were also removed from the analysis to exclude anomalous data. A sample of seven participants was required for a statistical power of >.80, and so even with these data exclusions Experiment 5 had sufficient statistical power to detect meaningful effects.

### Results

#### Foraging accuracy

**Fig. 6:**
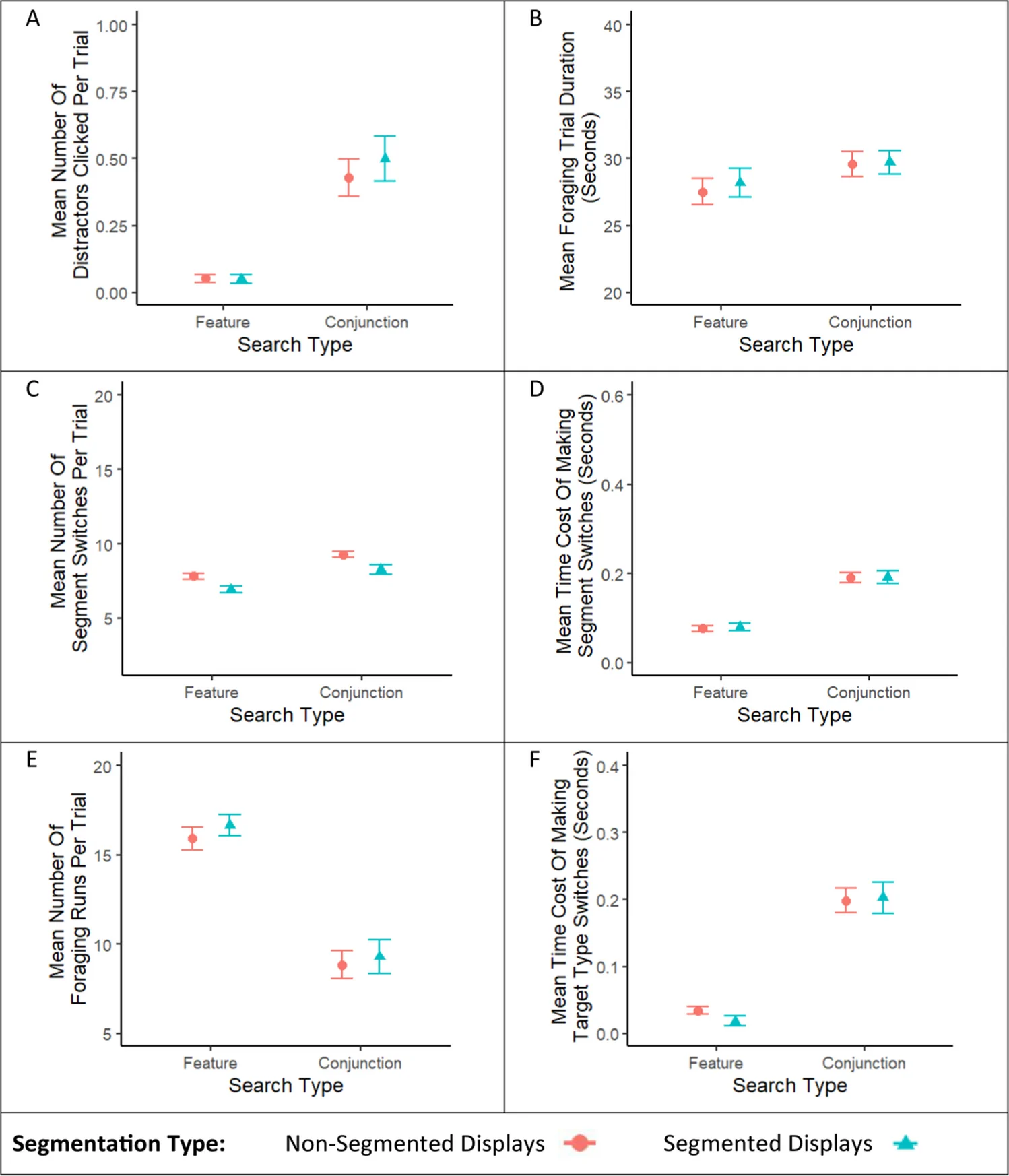
**A–F **Effects of search and segmentation type on (**A**) foraging accuracy, (**B**) foraging duration, (**C**) segment switches, (**D**) segment switch costs, (**E**) foraging runs*,* and (**F**) target type switch costs. Points represent mean values, and error bars represent standard errors

Trials excluded for containing errors were included in the analysis of foraging accuracy only. For foraging accuracy, there was a main effect of search type*, F*(1,91) = 60.04,* p <*.001, *η*_*p*_^*2*^ =.40, but not of segmentation type*, F*(1,91) =.42,* p =*.52, *η*_*p*_^*2*^ =.004, nor any interaction*, F*(1,91) =.40,* p =*.53, *η*_*p*_^*2*^ =.001. This measure did not meet the assumption of equal variances and so a Kruskal-Wallis test was conducted: *H*(3) = 45.5,* p <.*001. Significant differences were detected in post hoc comparisons of all pairings (*p*s <.001) except for between segmented and non-segmented display trials in feature trials and in conjunction trials (*p*s ≥.999). Additional Wilcoxon signed-rank tests were conducted that indicated foraging accuracy differed significantly in feature vs conjunction foraging trials completed within segmented and non-segmented displays (*p*s* <*.001). Overall, more distractors were selected during conjunction than feature foraging. These effects are shown in Fig. 6A.

#### Foraging duration^1^

For the measure of foraging duration (see Fig. 6B), an effect of search type was found*, F*(1,91) = 33.30,* p <*.001, *η*_*p*_^*2*^ =.27, such that foraging duration was longer in conjunction than feature trials. No effect of segmentation type*, F*(1,91) = 0.36,* p =*.55, *η*_*p*_^*2*^ <.001, nor interaction was found*, F*(1,91) = 3.03,* p =*.09, *η*_*p*_^*2*^ =.03.

#### Segment switches

For segment switches (see Fig. 6C), effects of search type*, F*(1,94) = 76.19,* p <*.001, *η*_*p*_^*2*^ =.45, and segmentation type*, F*(1,94) = 10.70,* p =*.001, *η*_*p*_^*2*^ =.10, were found, such that more segment switches were made in conjunction than feature foraging trials and fewer segment switches were made in trials with segmentation. These factors did not interact*, F*(1,94) =.23,* p =*.64, *η*_*p*_^*2*^ =.002. The measure of segment switch count did not meet the assumption of equal variances. Therefore, a Kruskal-Wallis test was conducted *H*(3) = 44.7,* p <.*001, with post hoc tests consistent with those of the ANOVA: all were significant (*p*s <.03) except for comparisons between segmented and non-segmented display trials in feature foraging *(p =*.2). Additional Wilcoxon signed-rank tests were conducted that indicated the number of segment switches made whilst foraging was significantly different between feature and conjunction foraging trials completed within segmented and non-segmented displays (*p*s* <*.001). More segment switches were made in conjunction foraging than in feature foraging trials. Segmentation therefore still impacted foraging by leading to fewer segment switches. More segment switches were also observed in conjunction than feature foraging.

#### Segment switch costs

For segment switch costs (see Fig. 6D), an effect of search type was found*, F*(1,93) = 76.70,* p <.*001, *η*_*p*_^*2*^ =.45, such that costs were greater during conjunction foraging than feature foraging. No effect of segmentation type was found*, F*(1,93) = 0,* p =*.96, *η*_*p*_^*2*^ <.001, nor was there an interaction*, F*(1,93) =.06,* p =*.80, *η*_*p*_^*2*^ <.001. As this measure did not meet the assumption of equal variances, a Kruskal-Wallis test was conducted H(3) = 62.29,* p* <.001. Post hoc tests were carried out and showed differences between all pairings (*p*s <.001) except for pairings between segmented and non-segmented displays in feature trials and in conjunction trials (*p*s ≥.999). Additional Wilcoxon signed-rank tests were conducted that indicated significantly differences in segment-switch costs made during feature versus conjunction foraging trials completed within segmented and non-segmented displays (*p*s* <*.001). Segment switch costs were greater in conjunction than feature trials.

#### Foraging runs

An effect of search type was found on foraging runs*, F*(1,94) = 178.88,* p <*.001, *η*_*p*_^*2*^ =.66, such that foraging runs were longer during conjunction than feature foraging. However, no effect was found of segmentation type*, F*(1,94) =.43,* p =*.51, *η*_*p*_^*2*^ <.001, nor was there an interaction*, F*(1,94) =.07,* p =*.79, *η*_*p*_^*2*^ <.001. The measure of foraging runs did not meet the assumption of equal variances and so a Kruskal-Wallis test was conducted, H(3) = 70.77,* p <.*001, with post hoc Dunn’s tests indicating differences detected between all pairs (*p*s <.001) except between segmented and non-segmented display trials in feature foraging and in conjunction foraging (*p*s >. 999). Additional Wilcoxon signed-rank tests were conducted and showed that the number of foraging runs made during a trial differed significantly between feature and conjunction foraging trials completed within segmented and non-segmented displays (*p*s* <*.001). More foraging runs were made during feature trials than conjunction foraging trials, as shown in Fig. 6E.

#### Target type switch costs

For target-type switch costs (see Fig. 6F), an effect was found of search type*, F*(1,93) = 107.23,* p <*.001, *η*_*p*_^*2*^ =.54, such that participants took longer to switch between the types of targets during conjunction than feature foraging. No effect of segmentation type was found*, F*(1,93) =.45,* p =*.50, *η*_*p*_^*2*^ =.005, nor interaction*, F*(1,93) =.03,* p =*.86, *η*_*p*_^*2*^ <.001. The measure of target-type switch costs did not meet the assumption of equal variances, and so a Kruskal-Wallis test was conducted H (3) = 80.33,* p <.*001. Post hoc Dunn’s tests indicated differences between all pairings (*p*s <.001), except for between non-segmented and segmented displays in feature trials and in conjunction trials (*p*s >.999). Additional Wilcoxon signed-rank tests were conducted that indicated the time costs of switching between target types was significantly different between feature and conjunction foraging trials completed within segmented and non-segmented displays (*p*s* <*.001). As before, target type switch costs were greater in conjunction trials than in feature trials.

### Discussion

The findings from Experiment 5 showed that effects of segmentation were reduced, or completely absent, when segmentation was only present at preview prior to the active foraging portion of the trial. Importantly, as in Experiment 4, segmentation no longer made foraging less optimal. Of the four foraging measures that segmentation impacted in Experiment 1 (error, foraging duration, segment switches, segment switch cost), only the number of segment switches was impacted by segmentation in Experiment 5. Even so, the impact of segmentation on segment switches was much reduced. In Experiment 1 the number of segment switches in trials with segmentation was typically 3–4, which was close to the minimum of three. In Experiment 5 the mean number of segment switches in segmented trials was around seven for feature foraging and around eight for conjunction foraging. As such, whilst segmentation did influence foraging behaviour, it was much less characterized by exhaustive foraging within a segment.

Experiments 4 and 5 therefore show that manipulating the visibility and availability of display segmentation appears to modulate the extent to which individuals structure their foraging. When the salience of the segmentation is reduced or when it is pre-viewed but not continuously presented alongside the stimuli, then several costs associated with segmentation are reduced or eliminated and the extent to which foraging is near exhaustive within segments is reduced. If use of the spatial structure was strategic, then we would not expect to see these reductions in the impact of segmentation. Segmentation was clearly visible in Experiment 4 albeit less salient, and in Experiment 5 pre-viewing a salient spatial structure should have enabled participants to use this to guide foraging; indeed, this was observed to an extent. As such, it seems likely that the presence of a salient spatial structure impacts foraging behaviour through bottom-up processes.

Nakashima and Yokosawa (2013) suggested that in their search task, a spatial structure may induce perceptual grouping. Perceptual grouping is often based on stimulus similarity (Duncan & Humphreys, 1989; Egeth et al., 1984; Guest & Lamberts, 2011) and thus is associated with changes in stimulus characteristics. As Nakashima and Yokokawa (2013) note, this is not the case when a spatial structure is imposed. However, in terms of theories of search such as Guided Search (Wolfe, 2021), it might be that a spatial structure acts to dampen activation in spatial regions of the saliency map that are currently unattended. The effect of this would be that foraging is more exhaustive within a segment, and this effect is magnified by the salience of the structure. Alternatively, it might be that the visual system represents boundaries between regions in such a way that makes them difficult to cross. For example, within a saliency map the boundaries might create peaks of activation, such that crossing these peaks to attend to items on the far side is effortful. Again, the impact of this would be to create more exhaustive foraging within a segment.

One of the central findings of Experiments 1–3 was that segmentation led to less-optimal foraging, with foraging either taking longer, or showing fewer targets selected within a given time frame. Moreover, even whilst the impact of segmentation was reduced in Experiments 4 and 5, Experiments 1–5 showed consistently that the spatial structure of foraging displays can influence individuals’ foraging organization even though there were no clear performance benefits of using spatial structure to organize foraging. As noted in Experiment 1, this is surprising as it runs counter to findings from visual search where inserting a spatial structure appeared to increase search efficiency (Nakashima & Yokosawa, 2013). It is also surprising insofar as a spatial structure appears to evoke a clear change in foraging behaviour that appears to be less efficient and so it is unclear as to why this behaviour was so persistent. A key consideration then is to consider how spatial structure might support foraging. When foraging through a display, it might be difficult to remember which areas have been foraged, leading to inefficiencies in foraging. This would particularly be the case in foraging tasks where the stimuli remain present when foraged. In such instances, use of spatial structure might be advantageous by supporting memory for which locations have been previously foraged. This is the focus of Experiment 6.

## Experiment 6

The aim of Experiment 6 was to examine whether spatial structure can be advantageous in foraging. A segment-by-segment approach to foraging combined with near-exhaustive foraging within a segment might be beneficial when cognitive load is high and keeping track of which locations have been previously foraged or rejected is difficult. In such situations, segmenting the display may enable the participant to focus their attention on a given segment, allowing for more accurate tracking of which areas have been foraged or rejected. Experiment 6 therefore increased the difficulty of the foraging task by leaving targets onscreen once they had been clicked (foraged). This meant that participants had no visual cues about where they had already foraged and so needed to rely on their memory or other systematic strategy in order not to return to previously found targets. If segmentation supports foraging under high memory load conditions, then it was expected that foraging would be more optimal, and that participants would less frequently return to areas they had already foraged when foraging segmented displays.

### Method

Experiment 6 was identical to Experiment 1, except that the targets no longer disappeared after being clicked. Note that targets briefly vanished for 150 ms as feedback for participants that their click was successful. Additionally, Experiment 6 also imposed a 20-s time limit on foraging as was the case in Experiments 3 and 4. This is because it was unfeasible to expect participants to repeatedly search displays that did not end until all targets had been selected at least once, otherwise these trials may be exceptionally long. A new metric was devised in Experiment 6 that measured the proportion of participants’ clicks in which a target that had already been selected was clicked on (within the entire display). This proportion of re-clicks thereby reflects how often participants returned to targets and regions they had already searched.

#### Sample

Experiment 6 was pre-registered (https://osf.io/k3jcs). A power analysis conducted in G*Power (Faul et al., 2007) estimated that a minimum of 52 participant data were needed for appropriate power. To compensate for using non-parametric analyses and the removal of individual trials, a target sample of 72 participants was set. A total of 130 participants were recruited for Experiment 6 which resulted in 72 participants who met the inclusion criteria outlined in Experiment 3. Again, participants whose data were excluded because they selected distractors in multiple trials across one condition were included in the analysis of foraging errors (distractor clicks) only. Participants (41 F, 29 M, two others) were aged 21–58 years (mean age = 39.79 years, *SD* = 10.35 years). They were recruited via Prolific.co and compensated £2.50 for their time. Post hoc power analysis conducted in G*Power (Faul et al., 2007) on the smallest significant effect size in Experiment 6 (*η*_*p*_^*2*^ =.06) found that a sample of 72 participants gave a statistical power ≥.999.

### Results

#### Foraging accuracy

**Fig. 7:**
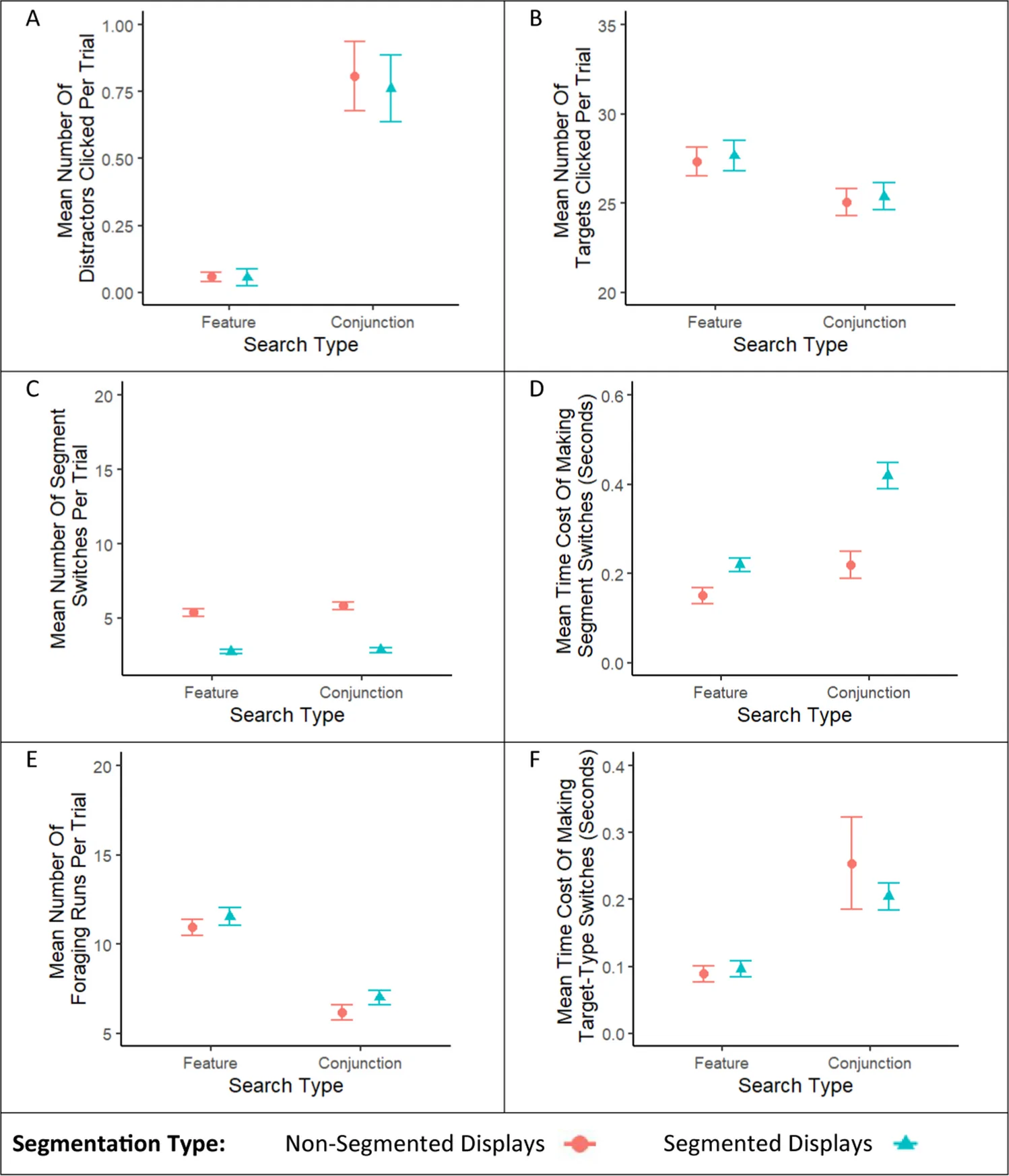
**A–F **Effects of search and segmentation type on (**A**) foraging accuracy, (**B**) number of targets selected, (**C**) segment switches, (**D**) segment switch costs, (**E**) foraging runs*,* and (**F**) target type switch costs. Points represent mean values, and error bars represent standard errors

As in previous experiments, trials and participant data excluded for containing distractor clicks were included in the analysis of foraging accuracy, and so the 58 additional participants’ data that were previously excluded from analysis (see above) were included in this analysis. There was an effect of search type*, F*(1, 129) = 55.45,* p* <.001, *η*_*p*_^*2*^ =.30, but not segmentation type*, F*(1,129) = 0.13,* p =*.72, *η*_*p*_^*2*^ =.001, nor interaction*, F*(1,129) =.09,* p =*.76, *η*_*p*_^*2*^ =.001. This measure did not meet the assumption of equal variances and so a Friedman ANOVA was conducted, which found significant differences between conditions: χ^2^_F_ (3) = 169,* p <*.001. All post hoc tests were significant (*p*s <.001), except for the comparison between trials involving segmented and non-segmented displays for feature foraging and for conjunction foraging (*p*s >.999). More errors were made during conjunction than feature foraging. These effects are shown in Fig. 7A.

#### Number of target clicks

For the number of targets selected (see Fig. 7B), a main effect of search type*, F*(1,71) = 86.49,* p* <.001, *η*_*p*_^*2*^ =.55 was found, but no effect of segmentation type*, F*(1,71) = 2.31,* p* =.13, *η*_*p*_^*2*^ =.03, nor interaction was found*, F*(1,71) = 0,* p =*.95, *η*_*p*_^*2*^ <.001. Fewer targets were selected during conjunction than feature foraging.

#### Segment switches

For segment switches (see Fig. 7C), there were effects of search type*, F*(1,71) = 4.81,* p =*.03, *η*_*p*_^*2*^ =.06, and segmentation type*, F*(1,71) = 272.71,* p <*.001, *η*_*p*_^*2*^ =.79, but no interaction*, F*(1,71) = 3.02,* p =.*09, *η*_*p*_^*2*^ =.04. The measure of segment switches did not meet the assumption of equal variances and so a Friedman ANOVA was conducted, χ^2^_F_ (3) = 157,* p <*.001. All post hoc comparisons were significant (*p*s <.05), except for the comparison between feature and conjunction trials involving segmented displays (*p >*.999) and involving non-segmented displays *(p =*.05). Segmenting the display reduced the number of segment switches.

#### Segment switch costs

For segment switch costs (see Fig. 7D), main effects were found for search type*, F*(1,67) = 63.18,* p <.*001, *η*_*p*_^*2*^ =.49, and segmentation type*, F*(1,67) = 52.75,* p <.*001, *η*_*p*_^*2*^ =.44, and an interaction was found between these factors*, F*(1,67) = 11.43,* p =*.001, *η*_*p*_^*2*^ =.15. Post hoc pairwise comparisons found differences between all pairings (*p*s ≤.001). This measure did not meet the assumption of equal variances and so a Friedman ANOVA was conducted, χ^2^_F_ (3) = 38.1,* p <*.001. All post hoc comparisons were significant (*p*s <.05), except for between segmented and non-segmented display trials in feature foraging *(p =*.87). Segment switch costs were greater when the display was segmented and in conjunction foraging relative to feature foraging. However, the impact of segmentation was observed in conjunction foraging tasks but not feature foraging tasks.

#### Foraging runs

For foraging runs (see Fig. 7E), effects of search type, *F(*1,71) = 105.10,* p <.*001, *η*_*p*_^*2*^ =.60, and segmentation type, *F(*1,71) =.8.16,* p =*.006, *η*_*p*_^*2*^ =.1, were found. More foraging runs were made during feature foraging than conjunction foraging, and more foraging runs were made in segmented display trials than non-segmented display trials. However, no interaction of search and segmentation types was found, *F(*1,71) =.24,* p =*.62, *η*_*p*_^*2*^ =.003.

#### Target type switch costs

For target type switch costs (see Fig. 7F), an effect of search type was found, *F(*1,65) =24.82,* p <.*001, *η*_*p*_^*2*^ =.28, but no effect of segmentation type, *F(*1,65) =.38,* p =*.54, *η*_*p*_^*2*^ =.01, nor interaction, *F*(1,65) =.58,* p =*.45, *η*_*p*_^*2*^ =.01. Target type switch costs were greater in conjunction foraging than in feature foraging.

#### Proportion of target clicks that were re-clicks

**Fig. 8:**
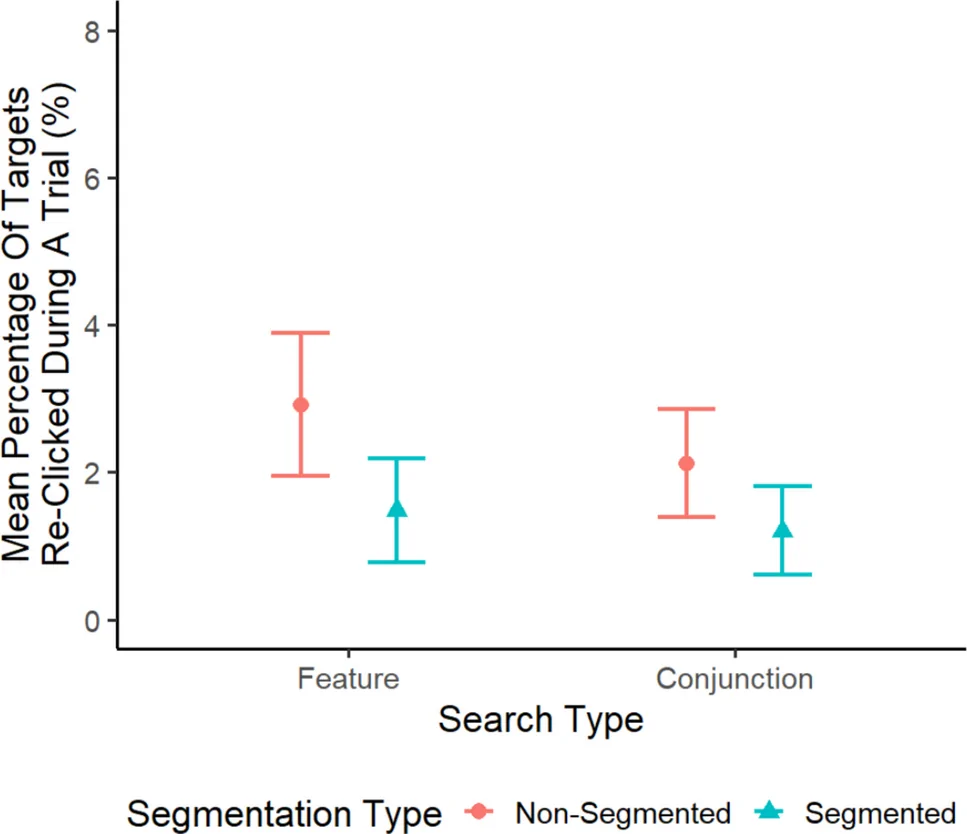
Effects of search and segmentation type on the number of re-clicked targets during a trial

For the proportion of target selections that were re-clicks on a target already selected by the participant, a main effect of segmentation type was found*, F*(1,71) = 10.29,* p* =.002, *η*_*p*_^*2*^ =.13. A greater proportion of target re-clicks were made when foraging non-segmented displays (mean =.03, *SD* =.07) than when foraging segmented ones (mean =.01, *SD* =.06). There was no effect of search type*, F*(1,71) = 1.98,* p* =.16, *η*_*p*_^*2*^ =.03, nor an interaction of segmentation and search types*, F*(1,71) =.45,* p* =.50, *η*_*p*_^*2*^ =.006. When the display was segmented, fewer targets were erroneously re-clicked, shown in Fig. 8.

### Discussion

Experiment 6 involved a more cognitively demanding task than Experiments 1–5, whereby individuals had 20 s to forage a display that did not provide any visual information about which items had previously been foraged. Individuals therefore needed to remember the location of targets they had selected or make use of some other strategy such as adopting a spatially systematic segment-by-segment strategy to avoid re-clicking targets they had already found. As in Experiments 1–5, segmentation clearly influenced foraging, with participants making far fewer segment switches. Indeed, the number of segment switches was similar to that observed in Experiment 3 (where the design was identical except for targets disappearing once clicked) and the number of segment switches in segmented displays was the lowest observed across all Experiments. This suggests that the combination of limited time and targets remaining present led to more foraging within a segment before switching a segment. Similar to Experiment 3, segmentation also increased the segment switch cost, and this was driven by conjunction foraging trials. Thus, participants found it harder to make a segment switch when using a segment-by-segment foraging approach where foraging was more difficult (conjunction foraging). Interestingly, the overall size of the segment switch cost appeared larger in Experiment 6 across all conditions compared to Experiment 3. This is perhaps to be expected. When targets do not disappear after being foraged, it will inevitably make it more difficult to choose to leave the area being foraged, as the decision to do so will include an additional assessment of whether the targets in that area have already been foraged. It is likely this that led to the inflated segment switch cost.

Whilst there was a clear impact of segmentation, unlike in Experiment 3, segmentation did not decrease search efficiency, with no difference in the amount of overall target clicks. Moreover, there was also a benefit of segmentation: as participants made fewer re-click selections of targets that they had already clicked in segmented displays than in non-segmented displays. As such, the segment-by-segment approach to foraging that appears to be induced in segmented displays is beneficial when foraged targets remain onscreen as this approach appears to enhance memory for which items have already been foraged. This is probably because segmenting displays allows participants to clearly mark the segments they have foraged, and thus use limited capacity resources to more accurately remember which targets within the segment they are foraging have already been foraged. It is possible this also facilitated the greater number of foraging runs observed when the display was segmented. Importantly, then, Experiments 1–3 appeared to show effects of segmentation that led to less optimal foraging that was only overcome when segmentation was less salient (Experiment 4) or not continuously visible in the foraging display (Experiment 5), Experiment 6 shows that segmentation can be beneficial, because the segment-by-segment foraging it induces can enhance memory for previously foraged locations.

## General discussion

Across six experiments, these experiments consistently show that spatial structure affects individuals’ adoption of spatially systematic strategies for foraging activity. Where displays were visibly divided into segments, individuals made fewer switches between display segments, compared to when displays contained no visible segmenting lines. This was true when displays were: presented as either segmented or non-segmented (Experiment 1), split into eight segments instead of four (Experiment 2), foraged under timed conditions (Experiments 3, 4, and 6) contained faintly visible segmentation (Experiment 4) or when segmentation was only visible during the trial preview (Experiment 5). Across the manipulations of Experiments 1–4, there were time costs to foraging segmented displays by their segments. A measured benefit of foraging by segments was observed in Experiment 6, whereby individuals made fewer erroneous target re-clicks in segmented displays compared to non-segmented displays. This indicates that spatial structure may support search by preventing return to previously searched areas.

In Experiments 1–6, individuals may have found it effortful to ignore the visual information presented by the spatial structure and therefore foraged in a segment-by-segment manner as a consequence of this. Even when time limits were imposed, individuals continued to forage displays by segments. It appears likely that bottom-up visual information from segmenting lines cannot be suppressed, rather than these effects being wholly driven by the adoption of systematic strategies. Whilst spatial structure continued to affect foraging when segmentation vanished or was made fainter, its effects on foraging efficiency were diminished. If foraging in a segment-by-segment manner was part of a robust and deliberate strategy, effects of segmentation on foraging should not diminish relative to the visibility or availability of structures. For example, in previous related work (Gilchrist & Harvey, 2006), some spatial systematicity was observed in the absence of any explicit visible spatial structure. However, in the work presented here, reducing the visibility or availability of segmenting lines did indeed affect the degree of spatial systematicity observed. Experiments 1–6 therefore show that the spatial structure of displays affects how they are foraged, likely via bottom-up processes as well as the potential for participants to adopt top-down spatially systematic strategies.

Experiments 1–6 replicate well-established effects of target types on foraging (Jóhannesson et al., 2016, 2017; Kristjansson et al., 2014; Ólafsdóttir et al., 2020; Thornton et al., 2019): conjunction foraging was consistently associated with slower and less-accurate foraging than feature foraging. Participants foraged for feature targets in shorter runs with faster switches between target types than in conjunction foraging, consistent with existing findings (Kristjánsson et al., 2014; Jóhannesson et al., 2017; Kristjánsson, Björnsson et al., 2020a, Kristjánsson, Thornton et al., 2020b). The present work adds to these findings by showing that these feature-conjunction foraging differences persist in a more challenging task whereby targets do not vanish after selection (Experiment 6). Furthermore, in some experiments the effects of segmentation appeared greatest in conjunction foraging, compared to feature foraging. These findings together suggest that spatial structure can increase the complexity of an already inefficient task such as conjunction foraging, by adding decision-making elements to the task.

Guided search theory (Wolfe, 2021) proposes that attention can be prioritized for areas of the visual display that are likely to contain targets. If one conceptualizes this theory to permit attention prioritization spreading across items within a segment, or equivalent local area of spatial structure, then it follows that individuals would be more likely to forage displays in a segment-by-segment manner. Another possibility is that the information from spatial structure may be represented in such a way that makes structural boundaries difficult to cross. For example, if such structures are particularly salient, then they may cause strong activations within attentional maps that are difficult to disregard. As well as inducing more spatially systematic search this may also contribute to the time cost in switching segments. Here we show for the first time that there is a cost associated with a segment switch and that this is highly reliable such that it appeared in all five experiments where foraging took place in displays with visible segments.

Previously it has been found that grouping items by spatial region can over-ride factors such as proximity (Palmer, 1992). In the work presented here, there was some sensitivity to structure, even in highly impoverished presentations of that structure, such as the short-lived visibility of the segmenting lines. The present results confirm the general finding that individuals show a tendency to prefer more spatially systematic patterns in visual search (Gilchrist & Harvey, 2006) and VR environmental exploration (De Lillo et al., 2014), as well as searching in clusters in similar tasks such as foraging for non-visible targets (Kerster & Kello, 2016; Kerster et al., 2016).

Foraging in a segment-by-segment manner may reduce the effort or perceived effort involved in the task under some conditions. The first potential candidate explanation is the fact that foraging within one segment at a time may place less demand on attention. If attention is only focussed within one segment, then this would effectively reduce the set size of the task at any given time since attention need operate only over one quarter of the total display at a time. The second candidate reason for adopting a strategy of foraging in a segment-by-segment manner is to reduce some of the demands placed on memory during foraging, thereby enabling memory to support foraging to a greater extent. Memory can support search during foraging in several ways (Shore & Klein, 2000). In foraging segmented displays, segmentation might aid spatial memory to encode the segments or general areas that have been searched. However, this potential benefit of segmentation may come with other costs. That is, constraining search to one segment at a time can result in individuals taking longer routes around the display and making more target-type switches to complete the task.

Experiment 6, unlike Experiments 1–5, showed a measurable benefit of using the spatial structure within a display to organise foraging behaviours. Displays in Experiment 6 contained no information about where had already been searched, as all targets remained visible even after selection. It is not feasible for an individual to remember which of the 80 items they had already attended to since memory for found targets and searched distractors is limited (Howard et al., 2011, 2020; Võ & Wolfe, 2015). Additionally, the capacity of inhibition of return, a mechanism proposed to limit attentional return to up to five areas and around 1,000 ms (Klein, 2000; Snyder & Kingstone, 2000; Wang & Klein, 2010) is insufficient to significantly help forage a display of 80 items. Another inhibitory mechanism that might potentially assist in these foraging tasks is visual marking (commonly demonstrated in preview display designs) but this is also highly capacity limited (Watson & Humphreys, 1997; Watson & Kunar, 2012). Spatial structure may therefore be beneficial if it allows the searcher to encode the identity of whole segments that have already been searched. Alternatively, spatial structure may facilitate the use of systematic search strategies such as row-by-row foraging routes within individual segments.

In six experiments manipulating the presence and availability of spatial structure in foraging displays triggered spatially systematic search. This was despite the spatial structure being uninformative about target locations and devoid of semantic or syntactic information. Such spatially systematic search may enable memory to better support foraging activity in terms of remembering which segments have been searched and may also reduce the effective set size by reducing the current search area to one segment at a time, making it feel like a useful strategy. This effect was strongest when segmenting lines were constantly present in the display and was even found when display segmentation was only visible at the start of a trial. However, using this strategy imposes an additional decision to switch segments which is demonstrated here to be associated with a time cost. Furthermore, spatially segmented search requires more target type switches to be made, which can introduce additional performance costs such as those historically associated with making target type switches (Jóhannesson et al., 2016; Kristjánsson, Björnsson et al., 2020a, Kristjánsson, Thornton et al., 2020b). Interestingly, however, the present findings did not consistently find that imposing segmentation onto displays led to increased foraging times. Therefore, other performance costs associated with segmentation may be in effect, such as traversing an increased distance around the display. Regardless of the costs associated with such a strategy, the presence of spatial structure in the display appears to encourage spatially systematic foraging.

## Conclusion

Many activities involve searching through highly structured environments: medical professionals search various imaging displays, CCTV operators search screens, sports players search the field of play for various targets and events. Understanding how structure affects these multiple-target searches can potentially allow for search activity to be better optimised to produce more successful results. Across six experiments, introducing spatial structure changed the dynamics of attention during this foraging task where participants search for multiple instances of feature and conjunction targets. Since this spatial structure is not informative as to the location of targets and contains neither semantic nor syntactic information, it sits outside of the framework of variables traditionally theorised to guide attention in the Guided Search model (Wolfe, 2021). Spatial structure appeared to trigger spatially systematic search strategies, increasing the tendency of participants to forage in a more segment-by-segment manner than they do in the absence of such structure. The spatial structure present here may have been sufficient to trigger a patch-based strategy whereby segments are treated as patches to be exploited for resources before moving onto the next patch, in the manner seen across a range of species (for a recent review, see Bella-Fernández et al., 2022). These six experiments suggest that spatial structure is a critical component of the environment that influences attention during foraging and deserves consideration in comprehensive explanations of search.

## Supplementary Information

Below is the link to the electronic supplementary material.

Supplementary file1 (DOCX 33 KB)

## Data Availability

The data and experimental task code for all experiments are available in the Open Science Framework repository (see above). Analysis code may be made available on request by directly contacting CH at the address given above.

## Footnotes

1 Note that in Experiment 5 trial durations did not include the time spent previewing displays, and trial durations were measured from the point that the first target was selected.

## Code availability

All analyses were conducted using R, which is freely available to individuals. Experiments were programmed using a mixture of python (PsychoPy) and JavaScript.
