# Supplementary material for: Spatial structure triggers systematic foraging: Segmenting search displays leads to searching by segments
Source: Atten Percept Psychophys. 2026 Jun 9;88(5):143. doi: 10.3758/s13414-026-03287-8 (PMC13249926; doi:10.3758/s13414-026-03287-8)
Supplement: Supplementary file 1 — Supplementary file1 (DOCX 33 KB) [file 13414_2026_3287_MOESM1_ESM.docx]

Experiment 1. Foraging for feature and conjunction targets in segmented (quartered) and non-segmented displays

|  | Mean number of distractors selected per trial | | Mean foraging trial duration (seconds) | | Mean number of segment switches per trial | |
| --- | --- | --- | --- | --- | --- | --- |
|  | Non-Segmented Display | Segmented Display | Non-Segmented Display | Segmented Display | Non-Segmented Display | Segmented Display |
| Feature Search | M = .05  SD = .14 | M = .08  SD = .20 | M = 28.26  SD = 5.84 | M = 28.66  SD = 6.48 | M = 7.72  SD = 1.44 | M = 3.72  SD = .96 |
| Conjunction Search | M = .31  SD = .45 | M = .47  SD = .63 | M = 31.82  SD = 6.41 | M = 32.67  SD = 6.15 | M = 9.63  SD =1.88 | M = 4.30  SD =1.47 |

|  | Mean time cost of making segment switches (seconds) | | Mean number of foraging runs per trial | | Mean time cost of making target-type switches per trial (seconds) | |
| --- | --- | --- | --- | --- | --- | --- |
|  | Non-Segmented Display | Segmented Display | Non-Segmented Display | Segmented Display | Non-Segmented Display | Segmented Display |
| Feature Search | M = .09  SD = .12 | M = .23  SD = .22 | M = 16.94  SD = 3.54 | M = 17.30  SD = 2.89 | M = .02  SD = .13 | M = .02  SD =.12 |
| Conjunction Search | M = .22  SD = .26 | M = .57  SD = .41 | M = 8.77  SD = 5.83 | M = 10.12  SD = 4.97 | M = .22  SD = .33 | M = .23  SD = .32 |

Experiment 2. Foraging for feature and conjunction targets in segmented (Eight-segment) and non-segmented displays

|  | Mean number of distractors selected per trial | | Mean foraging trial duration (seconds) | | Mean number of segment switches per trial | |
| --- | --- | --- | --- | --- | --- | --- |
|  | Non-Segmented Display | Segmented Display | Non-Segmented Display | Segmented Display | Non-Segmented Display | Segmented Display |
| Feature Search | M = .05  SD = .11 | M = .06  SD = .14 | M = 27.69  SD = 4.79 | M = 27.66  SD = 4.60 | M = 14.53  SD = 2.00 | M = 8.73  SD = 2.05 |
| Conjunction Search | M = .38  SD = .56 | M = .64  SD = .90 | M = 30.23  SD = 4.68 | M = 31.33  SD = 4.66 | M = 17.01  SD = 2.93 | M = 10.92  SD = 3.11 |

|  | Mean time cost of making segment switches (seconds) | | Mean number of foraging runs per trial | | Mean time cost of making target-type switches per trial (seconds) | |
| --- | --- | --- | --- | --- | --- | --- |
|  | Non-Segmented Display | Segmented Display | Non-Segmented Display | Segmented Display | Non-Segmented Display | Segmented Display |
| Feature Search | M = .10  SD = .10 | M = .17  SD = .14 | M = 15.79  SD = 4.84 | M = 17.36  SD = 4.04 | M = .03  SD = .14 | M = .03  SD = .15 |
| Conjunction Search | M = .19  SD = .17 | M = .32  SD = .21 | M = 8.24  SD =6.17 | M = 10.03  SD = 6.03 | M = .19  SD = .34 | M = .18  SD =.30 |

Experiment 3. Time limited foraging for feature and conjunction targets in segmented (quartered) and non-segmented displays.

|  | Mean number of distractors selected per trial | | Mean number of targets selected per trial | | Mean number of segment switches per trial | |
| --- | --- | --- | --- | --- | --- | --- |
|  | Non-Segmented Display | Segmented Display | Non-Segmented Display | Segmented Display | Non-Segmented Display | Segmented Display |
| Feature Search | M = .04  SD = .11 | M = .03  SD =.10 | M = 29.98  SD = 7.77 | M = 28.61  SD = 7.76 | M = 5.49  SD = 2.02 | M = 2.96  SD = 1.08 |
| Conjunction Search | M = .64  SD = .86 | M = .73  SD =.97 | M = 27.35  SD = 7.23 | M = 26.33  SD = 7.27 | M = 5.75  SD = 2.21 | M = 2.97  SD =1.39 |

|  | Mean time cost of making segment switches in seconds | | Mean number of foraging runs per trial | | Mean time cost of making target-type switches per trial (seconds) | |
| --- | --- | --- | --- | --- | --- | --- |
|  | Non-Segmented Display | Segmented Display | Non-Segmented Display | Segmented Display | Non-Segmented Display | Segmented Display |
| Feature Search | M = .11  SD =.21 | M = .17  SD = .28 | M = 12.4  SD =3.71 | M = 12.8  SD = 4.02 | M = .02  SD =.14 | M = .05  SD =.19 |
| Conjunction Search | M = .16  SD =.32 | M = .35  SD =.62 | M = 7.77  SD = 3.42 | M = 7.89  SD = 3.04 | M = .14  SD =.37 | M = .14  SD =.51 |

Experiment 4. Time limited foraging for feature and conjunction targets in faint segmented (quartered) and non-segmented displays.

|  | Mean number of distractors selected per trial | | Mean number of targets selected per trial | | Mean number of segment switches per trial | |
| --- | --- | --- | --- | --- | --- | --- |
|  | Non-Segmented Display | Segmented Display | Non-Segmented Display | Segmented Display | Non-Segmented Display | Segmented Display |
| Feature Search | M = .04  SD = .17 | M = .03  SD = .09 | M = 31.20  SD = 6.69 | M = 31.24  SD = 6.92 | M = 5.92  SD = 1.61 | M = 4.39  SD = 2.02 |
| Conjunction Search | M = .43  SD = .64 | M = .63  SD = 1.00 | M = 29.42  SD = 6.73 | M = 29.17  SD = 6.60 | M = 6.45  SD = 1.86 | M = 4.66  SD = 2.39 |

|  | Mean time cost of making segment switches in seconds | | Mean number of foraging runs per trial | | Mean time cost of making target-type switches per trial (seconds) | |
| --- | --- | --- | --- | --- | --- | --- |
|  | Non-Segmented Display | Segmented Display | Non-Segmented Display | Segmented Display | Non-Segmented Display | Segmented Display |
| Feature Search | M = .07  SD = .13 | M = .09  SD = .15 | M = 13.25  SD = 3.95 | M = 13.47  SD = 3.92 | M = .01  SD = .09 | M = .03  SD = .10 |
| Conjunction Search | M = .13  SD = .19 | M = .24  SD = .32 | M = 8.15  SD = 3.71 | M = 8.23  SD = 4.07 | M = .12  SD = .23 | M = .09  SD = .22 |

Experiment 5. Foraging for feature and conjunction targets in previewed-as-segmented (quartered) and non-segmented displays

|  | Mean number of distractors selected per trial | | Mean foraging trial duration (seconds) | | Mean number of segment switches per trial | |
| --- | --- | --- | --- | --- | --- | --- |
|  | Non-Segmented Display | Segmented Display | Non-Segmented Display | Segmented Display | Non-Segmented Display | Segmented Display |
| Feature Search | M = .05  SD = .11 | M = .05  SD = .10 | M = 27.51  SD = 7.28 | M = 28.19  SD = 6.95 | M = 7.81  SD = 1.47 | M = 6.92  SD = 1.54 |
| Conjunction Search | M = .43  SD = .51 | M = .50  SD = .53 | M = 29.61  SD = 6.81 | M = 29.72  SD = 5.60 | M = 9.29  SD = 1.48 | M = 8.26  SD = 1.98 |

|  | Mean time cost of making segment switches in seconds | | Mean number of foraging runs per trial | | Mean time cost of making target-type switches per trial (seconds) | |
| --- | --- | --- | --- | --- | --- | --- |
|  | Non-Segmented Display | Segmented Display | Non-Segmented Display | Segmented Display | Non-Segmented Display | Segmented Display |
| Feature Search | M = .08  SD = .14 | M = .08  SD = .15 | M = 15.92  SD = 4.66 | M = 16.66  SD = 3.73 | M = .03  SD = .13 | M = .02  SD = .13 |
| Conjunction Search | M = .19  SD = .21 | M = .19  SD = .22 | M = 8.85  SD = 5.68 | M = 9.29  SD = 5.97 | M = .20  SD = .33 | M = .20  SD = .35 |

Experiment 6. Time limited foraging for feature and conjunction targets in segmented (quartered) and non-segmented displays in which targets do not disappear after selection.

|  | Mean number of distractors selected per trial | | Mean number of targets selected per trial | | Mean number of segment switches per trial | |
| --- | --- | --- | --- | --- | --- | --- |
|  | Non-Segmented Display | Segmented Display | Non-Segmented Display | Segmented Display | Non-Segmented Display | Segmented Display |
| Feature Search | M = .06  SD = .19 | M = .06  SD = .35 | M = 27.35  SD = 6.80 | M = 27.66  SD = 7.22 | M = 5.37  SD = 2.11 | M = 2.76  SD = 1.08 |
| Conjunction Search | M = .81  SD =1.49 | M = .76  SD = 1.43 | M = 25.05  SD =6.40 | M = 25.37  SD =6.50 | M = 5.82  SD = 2.28 | M = 2.86  SD = 1.43 |

|  | Mean time cost of making segment switches in seconds | | Mean number of foraging runs per trial | | Mean time cost of making target-type switches per trial (seconds) | |
| --- | --- | --- | --- | --- | --- | --- |
|  | Non-Segmented Display | Segmented Display | Non-Segmented Display | Segmented Display | Non-Segmented Display | Segmented Display |
| Feature Search | M = .15  SD = .15 | M = .22  SD = .13 | M = 10.93  SD = 3.93 | M = 11.55  SD = 4.07 | M = .09  SD = .10 | M = .10  SD = .10 |
| Conjunction Search | M = .22  SD = .27 | M = .42  SD = .25 | M = 6.17  SD = 3.58 | M = 7.02  SD =3.43 | M = .25  SD = .56 | M = .21  SD = .16 |

|  | Mean proportion of targets re-clicked during a trial | |
| --- | --- | --- |
|  | Non-Segmented Display | Segmented Display |
| Feature Search | M = .03  SD = .08 | M = .01  SD = .06 |
| Conjunction Search | M = .02  SD = .06 | M = .01  SD = .05 |
